# Classifying and scoring of molecules with the NGN: new datasets, significance tests, and generalization

**DOI:** 10.1186/1471-2105-11-S8-S4

**Published:** 2010-10-26

**Authors:** Eddie YT Ma, Christopher JF Cameron, Stefan C Kremer

**Affiliations:** 1 Department of Biology at the University of Waterloo, Waterloo, Ontario, Canada; 2 School of Computer Science at the University of Guelph, Guelph, Ontario, Canada

## Abstract

This paper demonstrates how a Neural Grammar Network learns to classify and score molecules for a variety of tasks in chemistry and toxicology. In addition to a more detailed analysis on datasets previously studied, we introduce three new datasets (BBB, FXa, and toxicology) to show the generality of the approach. A new experimental methodology is developed and applied to both the new datasets as well as previously studied datasets. This methodology is rigorous and statistically grounded, and ultimately culminates in a Wilcoxon significance test that proves the effectiveness of the system. We further include a complete generalization of the specific technique to arbitrary grammars and datasets using a mathematical abstraction that allows researchers in different domains to apply the method to their own work.

**Background:**

Our work can be viewed as an alternative to existing methods to solve the quantitative structure-activity relationship (QSAR) problem. To this end, we review a number approaches both from a methodological and also a performance perspective. In addition to these approaches, we also examined a number of chemical properties that can be used by generic classifier systems, such as feed-forward artificial neural networks. In studying these approaches, we identified a set of interesting benchmark problem sets to which many of the above approaches had been applied. These included: ACE, AChE, AR, BBB, BZR, Cox2, DHFR, ER, FXa, GPB, Therm, and Thr. Finally, we developed our own benchmark set by collecting data on toxicology.

**Results:**

Our results show that our system performs better than, or comparatively to, the existing methods over a broad range of problem types. Our method does not require the expert knowledge that is necessary to apply the other methods to novel problems.

**Conclusions:**

We conclude that our success is due to the ability of our system to: 1) encode molecules losslessly before presentation to the learning system, and 2) leverage the design of molecular description languages to facilitate the identification of relevant structural attributes of the molecules over different problem domains.

## Background

In this section, we introduce the problem under consideration—the mathematical characterization of some observed biological characteristic over a set of molecules. We describe previous approaches to solving this problem—quantitative structure-activity relationship (QSAR) methods. Finally, we introduce a novel approach to solving the problem—using a formal grammar to structure an artificial neural network made up of re-usable components to process and learn the datasets.

### The problem of classifying and scoring molecules

The problem of QSAR is interesting for both its biomedical implications as well as its computational richness. The creation or discovery of a high fidelity generalizable approach that is capable of coping with a broad range of classification and regression problems promises to reduce the cost of drug development and to reduce the number of environmental toxins to screen. The computational step reduces lab testing. Finally, the problem leads to the development of innovative machine learning and statistical strategies. We can broadly separate the kinds of problems that we are interested in, into two categories: classification and regression. For classification problems the task revolves around identifying the membership of objects of interest, in classes of interest. By contrast, for regression problems a numerical score is given to the objects of interest. These two different approaches can be readily applied to the same types of problems depending on the desired result. For example, it is possible to classify molecules as toxic versus non-toxic (by some specific definition of toxicity), or alternately, it is also possible to describe the same set of molecules by a toxicity score. In this paper, we consider both problem classes, relying on the generality of the underlying artificial neural network model to be able to process the data.

In general, for either problem class, we are specifically interested in the problem of prediction; that is, to give an estimate for molecules whose actual properties are unknown. Thus, we are interested in our system’s ability to generalize to unseen examples. Our model (like all learning approaches), was built upon a training dataset of representative examples. In addition, and in order to evaluate the effectiveness of the system at generalization, we require a second test dataset which was not available to the system during the training process, but was used to assess its accuracy on previously unseen data. Thus, we typically divide our available data into training and testing sets. The details of this division are important with respect to the ability of the system to generalize and will be discussed in greater detail below.

In the next section, we discuss previous strategies to solve this problem.

### The classic approach (QSAR)

In this work, the NGN tackles the quantitative structure-activity relationship (QSAR) problem. The QSAR problem is defined as the development and use of machine learning methods to accurately and precisely classify and fit molecules based on some observed biological characteristic. These characteristics can relate to desired outcomes such as drug efficacy, drug bioavailability and pro-drug metabolism, or to undesired outcomes such as toxicity, mutagenicity and lethality. The classification task can be thought of as an easier case of the fitting (or regression) task, as the condition for classification is generally a binary threshold indicating a positive selection or a negative selection.

In a QSAR driven study, molecules must be represented so that a machine learning system can operate on them. Most machine learning systems operate on inputs that are vectors. So, typically, the first stage in any QSAR system is to encode the input data into real-valued or binary vectors. In this approach, the feature descriptor vector is selected such that each element in the vector describes what a domain expert considers a salient piece of information for a specific problem. In this work, we describe and compare our methods and results to those obtained in the literature. For classification experiments, three methods we compare against performed by Sutherland et al., (2003) [1] are Soft Independent Modeling by Class Analogy (SIMCA), Recursive Partitioning (RP), and Spline Fitting with a Genetic Algorithm (SFGA). Six methods we compare against reviewed by Li et al., (2005) [2] are Linear Regression (LR), Linear Discriminate Analysis (LDA), decision Tree (C4.5 DT), k-Nearest Neighbor (k-NN), Probabilistic Neural Network (PNN), and Support Vector Machine (SVM). Li et al. further uses Recursive Feature Elimination (RFE) to reduce the feature space and characterizes how this affects performance. Two related methods are compared against performed by Fountaine et al., (2005) [3]; they are Molecular Interaction Field (MIF-MIF) and Anchored-Molecular Interaction Field (A-MIF). Two other related methods are performed by Mohr et al., (2008) [4] where in molecular kernels based on anchored subgraphs are used; they are Molecular Kernels 1 and 2 (MK1, MK2). A Decision Forest approach is compared against, performed by Tong et al., (2004) [5]. For regression experiments, four methods reviewed by Sutherland et al., (2004) [6] are compared against, they are Comparative Molecular Field Analysis (CoMFA), Eigenvalue Analysis (EVA), Holo QSAR (HQSAR), and traditional 2D topology and 3D conforming descriptors (2.5D). Mohr’s molecular kernels are also compared in regression.

For support vector machine (SVM) approaches ([4], [7] and [8]), kernel operators are typically used in place of explicit input vectors, but the principle remains the same. The kernels reduce the input information to a simple (dot-product-equivalent) distance measure. Mohr’s work defined distance matrices based on the overlapping geometry that two molecules share anchored on triplets of bonded atoms. Cerioni and Ralaivola defined distances based on the occurrence and count of subgraphs between two molecules. It is important to recognize that these representations are lossy, in the sense that it is impossible to recover the original molecule from the feature vectors or kernel matrices generated. This method implies, that the feature vectors and kernel operators must be judiciously selected by domain experts in order to preserve the information that is salient to the problem to be solved, while they can (and ideally should) discard any irrelevant data or properties of the molecule. Naturally, this requires significant domain expertise on the part of the designer of the encoding mechanism. Not just chemical expertise in general, but also very problem specific expertise. The implications of this are three-fold. First, when tackling a novel QSAR problem, a domain expert is required to design an effective data encoding method. Second, any design remains problem specific and cannot, in general, be effectively applied to other problems (unless they are closely related). Third, any errors in the design of the encoding can result in the loss of useful information for the decision or output to be generated, and therefore can result in sub-optimal performance (even before considering the issues of learning, generalization, etc.). One final drawback to the use of descriptor vectors is that some of them are encoded in proprietary software packages so that their academic and commercial feasibility is bounded by third party contracts.

Our approach to the QSAR problem is dramatically different, in the sense that we do not encode the molecule as a vector. Instead, our representation is the molecular structure itself. By representing the structure directly, we are able to represent each molecule without loss of any information during the input process, thus providing our learning system with a maximum of information upon which to base its decision making or value predicting process. In this sense, the experimental approach that we use is most similar to other approaches that avoid using feature descriptor vectors entirely. A similar approach described by [9] uses a dynamic recurrent neural network. In this network, a traversal of the molecule is encoded such that atoms are hidden layers and bonds are their connecting weight layers. Many valid traversals may begin on each atom; however, only a single traversal is selected for each atom. To train weight matrices, the single traversals for each atom are all connected to one output root layer simultaneously. The accumulated activation value is the output for a given molecule in a QSAR problem.

### Our new concept

In contrast to [9], our method (outlined in the next section) uses a single structural representation of the molecules. This representation is based on a language for describing molecules by strings. The two formal languages we selected are SMILES [10] and InChI [11], although SMILES was used in the majority of experiments. Each of these languages describes single molecules with a single input string. The length and complexity of these strings are variable depending on the number of atoms in the molecule, as well as the amount of data intended to be encoded. In addition, the structure of the string is representative of the molecular structure.

The benefit of using this encoding is that domain expertise for each specific QSAR problem is not needed. The consistent generation of encoded strings represents a molecule’s topology, connectivity and optically active geometry losslessly. This benefit is in contrast to QSAR descriptor feature vectors which require the expert selection of specific features thought to be salient for individual problems and are also lossy. 

The next section details our approach and testing methodologies.

## Methods

In this section, we describe our model and the methods that we used to compare it to existing QSAR approaches.

Our method builds a model that computes an output value for a text string representation of a molecule. A specialized case of this model was previously introduced in [12,13]. It is the topology of the resulting parsed representation that allows the NGN to perform its computational tasks. We perform QSAR with this method. As mentioned earlier, this is similar to other graph traversal approaches, but is unique as the traversal produced is guided with a formal grammar. The NGN method operates on lossless string encodings that can be used to reconstruct the correct and unique topology of the molecule. By contrast, previous vector mapping approaches, which use only real-value or bit- vectors, are necessarily lossy. We are also capable of encoding complex structural configurations such as the expression of cis- and trans- bonds, chirality, and describe precisely where in a traversal particular charges or polar characteristics are found. Vector techniques are incapable of describing such location-specific features; and other graph traversal techniques do not document such complex structural characteristics.

In this section, we begin by describing textual molecular descriptions. These molecular strings are essentially one-line documents that describe a molecule as a visitor is traversing it from one atom to the next until all atoms and bonds have been visited.

Our computational model consists, at its core, of a deterministic LALR-1 parser and a feed forward back propagation neural network framework. The parser is used to structure the processing layers of the network; this is explained by an example and an abstract formalism in the following sub-sections.

We also identify and refine a set of benchmark problems for evaluating performance that others have used for QSAR approaches. We select a set of comparative techniques to which to compare our approach. And finally, we provide all the details of the parameters that we used.

**Figure 1:**
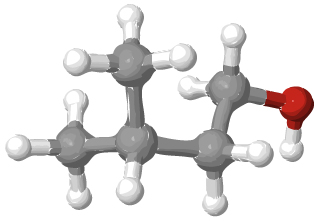
Artist’s conception of isopentanol molecule

Throughout this section we will work with a running example, namely the isopentanol molecule illustrated in Figure 1. The consistent use of this example facilitates the understanding of otherwise abstract topics.

### String representations of molecules

A number of string representations have been developed to describe molecules. The simplest and best known of these is the chemical formula. The chemical formula for our isopentanol example is C_5_H_11_OH. While this representation enumerates the atoms in the molecule it does not provide much information about the arrangement of the atoms in the molecule.

For our experiments, we leveraged two more informative representations, Simplified Molecular Input Line Entry Specification (SMILES) [10][14] and IUPAC International Chemical Identifier (InChI) [11]. These string formats were originally designed to address the need to uniquely key large databases of molecules. For each SMILES and InChI, the precise length of a string is determined by the number of atoms seen in a traversal of its corresponding molecule. The more atoms there are, the longer the string. A further combinatorics problem exists, as one could conceivably start on any atom and continue a traversal in any order. This is solved by using a consistent canonicalization algorithm for each dataset. Canonicalization constrains each molecule to a single unique string. Where available, the strings that document optical activity are used. When only a 3D conformer is known, the default canonicalization offered by OpenBabel was used. Each dataset was always treated to the same canonicalization. Three dimensional conformation information is also available and encoded into these strings for our experiments. Visually, SMILES strings distribute information about a molecular traversal uniformly across each string. InChI strings have particular substrings that contain different information; these substrings are called layers.

Isopentanol is represented by the SMILES string “CC(C)CCO”. Note that, in this representation, the parenthesized carbon atom indicates that this carbon is on a separate branch from the following two carbons and the oxygen. Hydrogen is not included in a SMILES representation, but can be inferred from the available valences. Thus, this representation provides more structural information than the simpler chemical formula.

A corresponding InChI string is “InChI=1S/C5H12O/c1-5(2)3-4-6/h5-6H,3-4H2,1-2H3”. In this representation, the first substring indicates that this string conforms to the first version of the InChI specification. Layers are delimited by slashes. The main layer is prefixed by ‘/’ and subsequent layers are prefixed by ‘/x’, where ‘x’ is a lowercase letter. Depending on the complexity of the molecule and the amount of information the user chooses to encode, the number of layers varies.

### Parsing

In order to use a string representation of a molecule, we must first parse the string. Parsing assigns a meaning to the symbols in the string and identifies a structural relationship between them using a set of grammar rules. The grammar rules for the SMILES language are defined in [10]. We have adapted these rules to make them suitable for processing with a LARL(1) parser. For the purpose of our explanation, we consider a small subset of the complete SMILES grammar rules. This subset is given in Table 1.

**Table 1:** Subset of SMILES grammar rules (derived from [10])

smiles	←	chain	1
chain	←	atom	2
chain	←	atom chain	3
chain	←	atom Nbranch chain	4
atom	←	C	5
atom	←	O	6
Nbranch	←	branch	7
branch	←	( chain_rparen	8
chain_rparen	←	chain rparen	9
rparen	←	)	10

The rules provide a system for rewriting a string of symbols. The start symbol for this rule set is “smiles”. The first rule states that the “smiles” symbol can be replace by the “chain” symbol. The second to fourth rules give three different ways of rewriting the “chain” symbol. This rewriting process is applied repeatedly until only terminal symbols are left. In this simplified language, all terminal symbols are a single character in length. By selecting from the set of possible rewrite rules for the full SMILES grammar, it is possible to construct any valid SMILES string.

For our example, we consider the derivation in Table 2, which shows how the string representing isopentanol is created from the start symbol “smiles”.

**Table 2:** Derivation of the SMILES string CC(C)CCO (Isopentanol) from the root smiles “symbol”

smiles	←	chain	Rule 1
	←	atom chain	Rule 3
	←	C chain	Rule 5
	←	C atom Nbranch chain	Rule 4
	←	C C Nbranch chain	Rule 5
	←	C C branch chain	Rule 7
	←	C C ( chain_rparen chain	Rule 8
	←	C C ( chain rparen chain	Rule 9
	←	C C ( atom rparen chain	Rule 2
	←	C C ( C rparen chain	Rule 5
	←	C C ( C ) chain	Rule 10
	←	C C ( C ) atom chain	Rule 3
	←	C C ( C ) C chain	Rule 5
	←	C C ( C ) C atom chain	Rule 3
	←	C C ( C ) C C chain	Rule 5
	←	C C ( C ) C C atom	Rule 2
	←	C C ( C ) C C O	Rule 6

**Figure 2:**
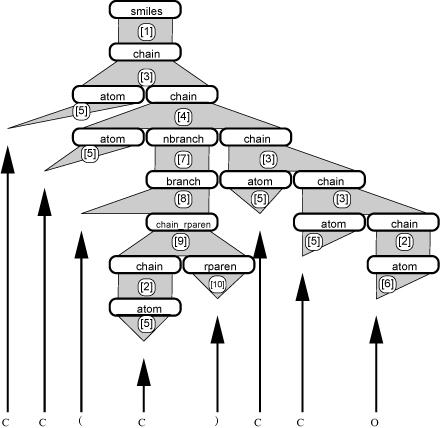
Parse tree for isopentanol

An alternate representation of this derivation is shown in Figure 2. In this figure, the parsing process is shown as a tree. Note that the rounded boxes indicate the rules applied at each step. This tree will form the basis of our model as outlined in the next section. We have designed our grammar to be unambiguous, which implies that for any string, there exists exactly one structural interpretation and, hence, one parse tree.

### Multi-layer neural network

**Figure 3:**
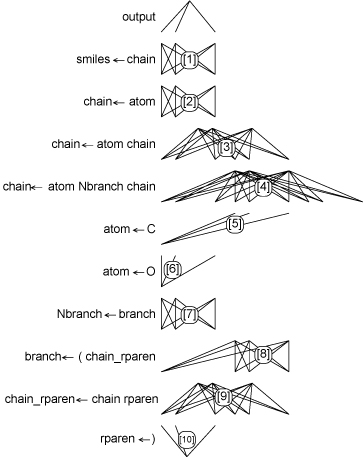
Weight layers for each rule given in Table 1

Rather than using a fixed neural network that operates on a constant number of features, like vector-based neural network solutions to this problem, our method dynamically constructs a custom neural network for each molecule to be considered based on the aforementioned parse tree. A larger molecule is thus represented losslessly by a larger parse tree and corresponding neural network. The dynamic network is constructed from a finite library of re-used, weight layers which persist between epochs of training and recognition tasks. These weight layers thus represent the accumulated training for the NGN system for each QSAR problem. The weight layers each correspond to a specific grammar rule and are assembled based on the string representation of the molecule and its parse. For our running example, Figure 3 depicts the re-usable weight layers for each rule given in Table 1. In this implementation, we decided to use 8 processing elements to represent each non-terminal symbol in the grammar (though in practise other numbers of elements can be used and need not be constant between non-terminal symbols). System parameters including the number of processing elements chosen were determined experimentally. We used only a single unit to represent each terminal symbol (an explanation of this decision follows later). Although not shown in the diagram, the weight layers also incorporate a set of bias terms at their terminal ends.

**Figure 4:**
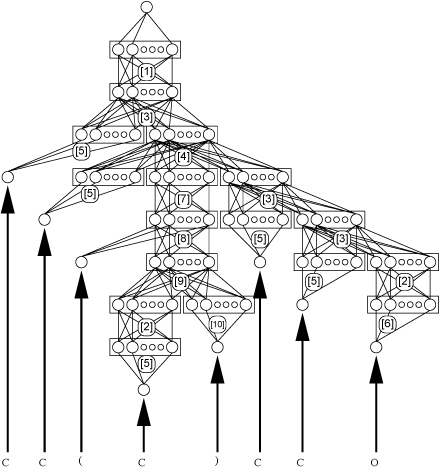
Neural grammar network for isopentanol

Having created these weight layers corresponding to the grammar rules, we can now construct a multi-layer neural network by substituting the grammar rules shown in Figure 2 by the appropriate weight matrices from Figure 3, and by replacing the terminal symbols in Figure 2 by individual output nodes. The result of this substitution is shown in Figure 4.

Having constructed the network in this way, we can now set the activation values of the input nodes (corresponding to the grammar terminal symbols) to values of 1.0. This may seem odd at first glance, since in most networks the input units represent the pattern to be processed, so setting them all to 1.0 would defeat that purpose. But in this case, the weight layers from these input units to the first hidden layers are actually specific to the input symbols that they represent, so the signal reaching these hidden layers encodes the presented symbols. (Another way to think about this is that the input symbols are represented in the signals sent to the first set of hidden values. In this interpretation, these input signals are themselves trainable.) From there, the structure of the network is itself an input signal (since it represents the molecule of interest).

We can now feedforward the activation values through all the processing elements of the network to the final output layer. At this point we can apply a standard error backpropagation algorithm to implement a gradient descent in error space just like with any other neural network.

The weight adjustment phase must be adapted slightly in order to accommodate the fact that the operating network is constructed with multiple instantiations of the same weight layers. In Figure 4, for example, the weight layers labelled [2] (occurring 2 times), [3] (occurring 3 times), and [5] (occurring 5 times) are the same weight layers. Fortunately, a correct gradient can be realized simply by summing the weight changes for each weight layer as computed by the normal gradient descent method. That is, the total change ∆*W_ij_* to one of the weights (from *j* to *i*) that corresponds to (e.g.) grammar rule [3] would be given by

 (1)

where  and  are the weight changes computed for the first, second and third instantiations of the [3] weight layer, respectively. These individual weight updates can then be computed as:

 (2)

in the notation of [15]. (Of course, advanced training methods like momentum, etc. can also be added.). Finally, it is important to note that the weights are updated in the weight layer library and that subsequent molecules will be constructed from these updated weight layers and themselves cause the weight layers to be further updated throughout the training process.

### NGN math

While we first presented a specific version of this approach in [12], our method is, in fact, much more general than one specific representation language or problem domain. In this paper, we abstract the example from the specific case presented above, and describe how an NGN would be used to process strings from an arbitrary grammar, *G*. In our work we have studied both SMILES and InChI, but other grammars are possible as well. We extend our approach to an arbitrary grammar and problem domain using a mathematical notation described below. We specifically discuss elements of graph theory, formal languages and recurrent neural networks and how they compose the NGN. Formal language strings must be parsed deterministically, and without ambiguity or conflicts.

We utilize the below notation to indicate an input string.

Input strings,* s*(*t*), are a concatenation,* C*, of input tokens, *s*(*t*)*_i_*. This concatenation operator,* C*, will be seen again when we map internal symbols of the grammar to hidden layers of the NGN.

The parse trees that arise (e.g. Figure 2) form the graph on which the neural network layers (e.g. Figure 3.) of the NGN will fit (e.g. Figure 4.). We define the characteristics required for a formal grammar to fit the NGN structure.

*G* =<*V*,Σ,*s*,*P*>

A grammar (*G*) of a formal language is a four tuple of internal symbols (*V*), terminal symbols (Σ), a start symbol (*s*|*s* ∈* V*) and production rules (*P*) that map a left-hand internal symbol to a sequence of right-hand symbols. Right-hand symbols can be solely terminal symbols or a mixture of internal and terminal symbols. For simplicity, we impose in this discussion that the right-hand of a production rule must be either a concatenation of internal symbols, or a single terminal symbol (<*V*^+^, Σ >). We can now consider parsing to be the activity of constructing a unique graph driven by an input string and grammar of a formal language. We stipulate that this graph is rooted, directed and acyclic which is a more strict type of tree. An input string *s*(*t*) has a corresponding parse tree (*T*(*t*)) at time *t*. The time variable allows us to index each successive string and its tree distinctly. In this work, each index corresponds to a different molecule, but our model is far more general and can be readily applied to different problem domains.

The tree* T*(*t*) at time* t* is composed of a set of nodes* N*(*t*) and sequences of edges  where a single edge is an ordered pair of nodes . We stipulate that our tree is strictly composed of sequences of edges rather than edges alone as it facilitates mapping sequences of internal nodes to symbols of the grammar. Directions are defined such that the root has only inbound edges. For a pair of nodes (*N_a_*(*t*),*N_b_*(*t*)) connected by *E*_(_*_a,b_*_)_(*t*) from* a* to* b*, *N_b_*(*t*) is the parent, and* N_a_*(*t*) is the child. From the tree, we map a sequence of edges  from a parent to its sequence of children onto the production rules* P* of the grammar* G* defined earlier. This is done such that the left-hand internal symbol maps to the parent node and that the right-hand sequence of symbols maps to the sequence of child nodes. We keep the sequence consistent so that each edge* E* uniquely connects the left-hand symbol to a specific right-hand symbol in sequence.

An artificial neural network is composed of alternating activation layers and weight layers when considered vertically. The NGN utilizes this same general architecture. The node layers are each composed of a real-value activation vector. The weight layers (*W*) that we discuss are each composed of a weight matrix (*w*) and bias vector . We map a unique sequence of weight layers onto each production rule of a given formal grammar* G.* A weight layer corresponds to an edge* E.* This edge* E* maps to the left-hand symbol of a* p* and a specific symbol on the right-hand side. The weight layer selected is thus the one that is indexed given the left-hand and right-hand symbols of the specified production rule *p*. If we discuss two node layers joined by a weight layer, the parent layer is the output layer and the child layer is the input layer. A weight layer,* W*, that occurs in an NGN will occur as many times as the corresponding production rule, *p* ∈ *P*. If data is processed more than once by a weight layer,* W*, in a neural network topology, then that topology is said to be recurrent. This is often the case in the grammar defined for molecular traversals in the current work. To clarify, each production rule (*p* ∈* P*) has its own corresponding weight layers* W_p_*(*t*), one for each right-hand symbol. This is indexed by time step as the values in each* w* and each  are tuned during NGN training.

Node layers that are not input layers (graph leaf nodes) or the final output layer (the single graph root node) are hidden layers. Hidden layers contain the activation vectors (*α_i_*) which hold the intermediate calculation of the network. These hidden layers are placed into the NGN topology corresponding to the left-hand side of production rules i.e. each to a single internal grammar symbol (*V*). The production rule defines precisely which weight layer should be used to connect two symbols in the grammar. When we consider a production rule as a whole, it is necessary to connect a sequence of edges  from the left-hand symbol to each right-hand symbol. When we do this, the identified weight layers are placed into the NGN and appropriately connected to the node layers mapped to each grammatical symbol of the right-hand sequence of symbols.

When alternative deterministic parses are defined by the grammar, node layers corresponding to right-hand symbols that are connected propagate activated values as expected. These symbols are connected because they are part of the current parse. Those symbols that are not connected are substituted by an activation vector flushed with zero values (0.0) for each node of the layer.

Input layers are node layers that have no child layers (nor corresponding bridging weight layers). The input string *s*(*t*) is lexicographically broken apart, and mapped to the terminal symbols (Σ) of the grammar (*G*). The input layer receives input depending on what production rule a weight layer is associated with and also the input string *s*(*t*). As tokens from the input string, *s*(*t*)*_i_*, are recognized, corresponding length-one vectors with an activation value of (1.0) are instantiated and placed onto the NGN topology as input layers. The production rule (*p* ∈ *P*) responsible for recognizing the input token may support more than one character to admit as input. In this work, this can be a *W_p_* which accepts chemical element symbols or a *W_p_* that accepts single digit integers etc.. In the latter case, that production rule corresponds to a weight layer *W* that has only as many nodes (processing units) as there are tokens it is capable of accepting. The activated length-one vector which indicates the identified token to the NGN is then connected to a specific node of the weight layer as each node of the weight layer is indexed exactly as the sequence given by the production rule *p*. The remaining inactive nodes receive a value (0.0) as input. In the graph topology, these input layers correspond to the leaf nodes.

The NGN topology has been described using a specific case of a tree, being rooted and directed with the root as a universal directional sink. We described dynamic parts of the NGN that change due to different conditions. The storage associated with each weight layer is permanent and is each associated with an edge connecting a pair of left- and right-hand symbols for each grammatical production rule. The topology of the NGN changes for each exemplar string, and is uniquely determined by the parse tree constructed. The values of each weight layer change during training, and the values of the activation vectors in each node layer change representing transient calculations. As the grammar and its corresponding weight layers persist throughout training, each weight layer is reused whenever the same grammar rule is called upon. Each weight layer is also reused if different strings in the dataset are parsed with the same productions. This reuse is where the NGN gains its uniqueness and power as a syntax-dependent string recognition device. A finite computational model is capable or processing inputs of unbounded length. The collection of weight layers represents the accumulated training that the NGN system has seen.

How NGN topology is derived has been established. We use this framework to now describe how calculation is performed.

The number of processing units (nodes) of a node layer can be arbitrarily defined by the end user (in all of our examples, we used 8 nodes per layer).

The feed forward algorithm is defined here. Aside from topology and the possibility of recurrence with repeated use of the same weight layer, the NGN system does not differ from the familiar feed forward algorithm used for artificial neural networks. This is a formalization.

The function **activity** is defined as follows, and produces the activation for a single hidden layer (internal node) of the NGN tree.

Given the parse tree, the NGN is traversed in breadth first order so that the {1.0} and {0.0} values of the input layers are computed first against the respective weights of their production rule edges, and the root (output layer) is computed last. In the above, we first concatenate all of the weight layers* w* involved with a single production rule* p* ∈* P.* For a production rule, these weight layers are indexed by their left-hand parent internal grammar symbol (*u*) and child (*v_i_*) for a specific right-hand grammar symbol. The activations  are those of each child layer, and the operator × is matrix multiplication. The bias vector  (a part of the weight layer) is then added to each value of the net vector. The net vector is then transformed by the logistic function to normalize the activation values to the range (0.0, 1.0).

The logistic transfer function **L** as we have defined it for vectors is:

With the normalization of the current activation, the sibling or the parent of this hidden layer is processed next. This traversal continues until all activations are calculated given their respective weight layers and a final output vector is retrieved from the root node of the NGN tree.

To tune weights, gradient descent with back propagation of errors is used. Tuning weights requires three steps. First, calculation of the error gradient is done; second, the amount that each weight must be changed is calculated; third, the weights are updated. The error gradient calculation is performed on a breadth first traversal starting at the root layer; the delta is performed on a depth first traversal starting at the leaves. Finally, weight update can be performed in either breadth or depth first.

The function **gradient** is defined as follows for the output layer; it calculates the error gradient from the output vector to the desired target output.

The function **gradient** is defined as follows for each hidden layer; it calculates the error gradient for each activation vector and finds the correct direction to tune weight layers.

The  vectors are the gradients of the net activation to a processing unit with respect to the weight under consideration. A node layer (output layer or hidden layer) is indexed as *v* and its parent is indexed as *u*. The values of the vectors and matrices are indexed using *j*, the index of a node in a layer and *i*, the index of a node in its parent’s layer (there are *j* many nodes in layer *v* (child) and *i* many nodes in layer *u* (parent)). The output layer’s gradient, , is calculated from its own activation, , and the target pattern . A hidden layer’s gradient  is computed from its own activation , its parent’s activation  and its parent’s gradient . Weight layers are only adjusted when they bridge between two node layers. Notice that there is no weight matrix in the former equation to bridge between the output and target vectors. In the latter equation, the summation indicates that the gradient of each node, , in a layer, *v*, is to be calculated with respect to an index *j* of corresponding weight values in the weight matrix, ; that is, all of the values corresponding to the parent layer, *u*, indexed by *i*, contribute to a single value, , in the gradient vector, .

Now that gradients have been calculated in a depth first traversal, we can calculate the changes to be made for each weight layer’s weight matrix, ∆*w*, and bias vector, , in a breadth first traversal.

Notice that the above formulas correspond to time steps (*t*). Each step in the breadth first traversal is considered a time step. For clarity, we liberally interchange the words ‘change’ and ‘delta’ to mean the same thing. The first formula describes the change in weight layer (∆*w*) corresponding to a node’s parent and itself (*u* ← *v*). A user tunable parameter, the training constant (*η*) defines how much change is to occur based on the error  at this time step; this is calculated against the activation vector  of the node layer *v*. The momentum coefficient (*α*), also user tunable, defines how much change to carry over from (∆*w*) of the previous time step (*t* − 1). The indices* j* and* i* correspond to the neural network processing nodes for a layer’s parent and itself respectively. In the second formula, the change in bias  is calculated. In contrast to calculating the weight matrix change, the change in bias is calculated with only information that pertains to the node’s parent layer.

Finally, a weight update is performed. While the particular traversal is arbitrary, we chose to perform weight update in a depth first pass. A weight update is given by summing each of the weight layer changes with each of their corresponding weight layers. This includes the changes needed for each of the weight matrices and the bias vectors. Recall that since weight layers may exist more than once within a NGN topology, the input string and grammar rules may define a parse structure that is recursive. This means that a single weight layer will be summed against more than one weight change matrix. It is of course possible that particular symbols do not exist at all in a given parse. When this happens, a weight layer simply is not updated given the current string. This three-phase back propagation training is performed once for each exemplar in every epoch of training.

### Dataset selection

In this work, we compared the performance of our technique against other QSAR approaches that are found in the literature. To this end, we have evaluated a broad range of feature vectors and a diverse set of QSAR problems. The first set of experiments describe the NGN used for the classification task. The second set of experiments approach the regression task. Finally, the third set of experiments are regression trials performed on a dataset that we have designed.

In the first suite of experiments conducted we compare our method against those published in literature for the classification of molecules into bioactive or inactive molecules. We first compared with a suite of machine learning methods reviewed by Sutherland et al., (2003) [1]; soft independent modeling by class analogy (SIMCA), recursive partitioning (RP) and spline fitting with a genetic algorithm (SFGA). These techniques are used in classification trials for separating bioactive and inactive molecules for the datasets [1] dihydrofolate reductase inhibitors (DHFR), benzodiazepine receptor binders (BZR), cyclooxygenase-2 inhibitors (Cox2). We then compared our results against the machine learning methods reviewed by Li et al., (2005) [2] linear regression (LR), linear discriminate analysis (LDA), decision tree (C4.5 DT), k-nearest neighbours (k-NN), probabilistic neural networks (PNN) and support vector machines (SVM). These methods were used for the classification of molecules that can or cannot cross the blood brain barrier (BBB) [2]. Two methods described by Fountaine et al., (2005) [3], molecular interaction fields (MIF) and molecular kernel (MK) are compared against in the factor Xa inhibitors (FXa) dataset [3] classification. The final experiments conducted were the classification of molecules into estrogen receptor binders (ER) [16][17] and androgen receptor binders (AR) [18]; these two datasets were retrieved from the Endocrine Disrupter Knowledge Base (EDKB). We also report on the internal cross validation experiment performed on the ER dataset by Tong et al., (2004) [5].

The second suite of experiments conducted involved comparing the regression performance of the NGN with that of other published techniques. This was done for the datasets reviewed by Sutherland et al., (2004) [6]; they are glycogen phosphorylase B inhibitors (GPB), angiotensin converting enzyme inhibitors (ACE), acetylcholinesterase inhibitors (AChE), cyclooxygenase inhibitors (Cox2), thermolysin inhibitors (Therm), benzodiazepine inhibitors (BZR), thrombin inhibitors (Thr), dihydrofolate reductase inhibitors (DHFR). Our approach was compared against methods [6] for descriptor generation as follows comparative field analysis (CoMFA), eigenvalue analysis (EVA), holographic QSAR (HQSAR) and descriptor vectors describing a hybrid of 2D topology and 3D conformation information (2.5D); and finally, the molecular kernel methods mentioned previously (MK1 and MK2) [4].

By comparing our results to previously published work on the same datasets, we can be assured that we are comparing against effective implementations and configurations of competing approaches, and that these methods were applied by experts in their use.

The final suite of experiments reviewed involves a new dataset that we have introduced recently [19]. The objective of using this dataset was to apply the same techniques to a novel problem set which we had not anticipated when designing the system. We did this to evaluate the flexibility of our approach.

Unfortunately, because the dataset is new, we cannot compare our results to those of other experts applying their favoured techniques to the same data. As a compromise, we have compared our results to those obtained using a number of popular molecular descriptors and a vanilla neural network. While we do not claim that the neural network represents a state of the art technique, it gives a rough indication of the performance that can be expected from alternative machine learning methods that use lossy descriptors. Our dataset consists of molecules to be classified based on their toxicity and their target organ of interaction for the mouse and rat species.

## Results and discussion

### NGN in QSAR classification experiments

In this section we discuss classification experiments, the first suite of experiments performed with the NGN. We first describe the datasets selected, then the experimental design and parameters, and finally the results for these experiments.

Datasets we selected must meet these criteria: they must be freely accessible in the literature, have a sufficient number of exemplars for QSAR, their three dimensional conformations must also be solved and available, and results of previous attempts to apply QSAR must be reported in the literature. A total of seven datasets are used in classification experiments, and ten for regression.

We conducted two kinds of classification and regression experiments. The first kind is an internal cross validation wherein a dataset is broken into approximately even groups such that one group is used as the test set while the remaining groups are used as the training set. We refer to this as a Leave-20%-Out internal cross validation design for classification and as a Leave-5%-Out internal cross validation design for regression. The Leave-20%-Out partition size for classification is adopted based on previous work conducted for the sake of comparison while the Leave-5%-Out partition size is chosen as the datasets for regression are of a smaller size on average. For classification, each group is used as a test set ten times for a total of fifty trials. These are referred to as internal cross validation experiments as a given dataset is broken apart into partitions without the use of an external dataset; this is in contrast to the designed test set strategy where a training set and testing set are explicitly designed. The second kind of experiment is based on designed test sets provided by the studies where we obtained the datasets. Some of these designed sets aim to maximize dissimilarity between molecules of a test set thereby testing the extrapolation power of the QSAR method analyzed. For each designed test set, we ran ten trials total. Table 3 summarizes the parameters we used for the both kinds of classification experiment, the Leave-5%-Out cross validation design for regression and the designed test sets for regression. The training constant* η* and momentum coefficient* α* have been discussed in the methods section. Convergence during training is determined by the root mean squared error dropping below a certain arbitrary threshold (RMSE). Weight matrices and bias vectors have random initial values; notice that we use larger values with magnitude in [1.0, 1.6] on either side of zero. We found this enabled convergence in a more reasonable number of epochs without sacrificing the accuracy of system predictions. For classification, we found a drop down to 5000 epochs to convergence; for regression, 7500 on average. The maximum number of epochs indicates when the system restarts having given up. The number of hidden nodes used in the neural network can be tuned for each grammatical symbol, but we determined that a global value of 8 for each SMILES and InChI was appropriate. The output scale is the range we have normalized the target output to accommodate the logistic transfer function used in the neural network computations. These values were derived experimentally and balance the training time against any loss in accuracy.

**Table 3:** A summary of the final experimental parameters used in both classification experimental designs, in regression Leave-5%-Out internal cross validation, and in designed regression test sets.

Parameter	Classification	Regression 5%-CV	Regression Designed
Training Constant (*η*)	0.60	0.30	0.33
Momentum Coefficient (*α*)	0.90	0.10	0.66
Convergence Threshold (**RMSE**)	0.05	0.03	0.04
Initial Random Weight Values	[−1.6, −1.0]	[−1.6, −1.0]	[−1.2, −0.4]
	∪ [1.0, 1.6]	∪ [1.0, 1.6]	∪ [0.4, 1.2]
Maximum Number of Epochs	5000	7500	7500
SMILES-NGN Hidden Nodes	8	8	8
InChI-NGN Hidden Nodes	8	8	8
Output Scale	(0.2, 0.8)	[0.2, 0.8]	[0.2, 0.8]

Table 4 summarizes the datasets used in the classification experiments, we describe how the datasets are broken down into positive classifiers and negative classifiers given their ranges of activity and a threshold. The ranges for each dataset are expressed in a logarithmic scale and are shown in the table. The scales are either *p* for negative base-ten logarithm, or log for base-ten logarithm. The values correspond to IC_50_ indicating the required concentration such that half of the target protein or receptor is bound or inhibited or they correspond to *K_i_*, a ratio of concentrations, or they correspond to relative binding affinity against (RBA). Of exception are the blood brain barrier (BBB) and factor Xa (FXa) datasets. The BBB dataset is broken up such that molecules that exist at 10% concentration on the brain side of the barrier are considered penetrators (positive). The FXa dataset has a wide threshold such that molecules that have an affinity of* K_i_* > 1*µM* are negative binders, and those that have* K_i_* ≤ 10*nM* are positive binders.

**Table 4:** A summary of the datasets used in classification experiments. † The range and threshold information was not provided in [2] and [3] for the BBB and FXa datasets, respectively.

Dataset	Dataset Full Name	Size	***N*** **^+^**	***N*** **^−^**	Range	***θ***	Units	Reference
**AR**	Androgen Receptor	202	146	56	[<−3.56, 2.27]	−3.56	logRBA	[24][18]
**BBB**	Blood Brain Barrier	415	276	139	†	†	pK*_i_*	[2]
**BZR**	Benzodiazepine Receptor	405	230	175	[<4.2, 9.5]	7.0	pIC_50_	[1]
**Cox2**	Cyclooxygenase 2	467	273	194	[<4.0, 9.0]	6.5	pIC_50_	[1][25]
**DHFR**	Dihydrofolate Reductase	756	302	454	[<3.0, 10.5]	6.0	pIC_50_	[1]
**ER**	Estrogen Receptor	232	131	101	[<−4.50, 2.60]	−4.50	logRBA	[24][17][16]
**FXa**	Factor Xa	435	279	156	†	†	pK*_i_*	[3]

In order to evaluate performance, we used several values gleaned from the literature. Concordance *Q* indicates the overall ratio of correct classifications performed by a system for a given dataset. Sensitivity *SE* is a ratio of correct positive guesses over all exemplars known to be positive. Specificity *SP* is a ratio of correct negative guesses over all exemplars known to be negative. Finally Matthew’s Correlation Coefficient *MCC* is used as a value that attempts to reward a system that behaves well for each *SE* and *SP* in concert so that a system that exceeds in one value but is lacking in the other scores poorly overall. Each of these values has a range [0.0, 1.0] such that 1.0 represents perfect performance.

The NGN performs variably when operating with the first three datasets (BZR, Cox2, DHFR) as seen in Table 5, Table 6, Table 7 given by Sutherland et al., (2003). The NGN performs the best given the DHFR dataset for both internal cross validation as well as overall.

**Table 5:** A summary of the performance on the **BZR** dataset with results as reported by [1] compared to the InChI-NGN in this work. Missing MCC values in the table reflect missing information in the primary literature.

Design	Method	***Q*** **(%)**	SE(%)	SP(%)	MCC
40% Test Set	SIMCA	72	68	76	-
	RP	69	64	74	-
	SFGA	75.5	70	81	-
	InChI-NGN	63.2	62.1	64.6	0.265

Leave-20%-Out	SIMCA	71.5±11.0	73±10	70±12	-
	RP	65.5±12	68±12	65±12	-
	SFGA	68.5±12	69±11	68±13	-
	InChI-NGN	69.9±1.98	73.4±1.87	65.3±2.29	0.387±0.041

**Table 6:** A summary of the performance on the **Cox2** dataset with results as reported by [1] compared to the InChI-NGN in this work. Missing MCC values in the table reflect missing information in the primary literature.

Design	Method	***Q*** **(%)**	SE(%)	SP(%)	MCC
40% Test Set	SIMCA	71	75	67	-
	RP	71	79	63	-
	SFGA	73.5	75	72	-
	InChI-NGN	65.1	62.5	68.4	0.307

Leave-20%-Out	SIMCA	78±9	79±9	77±9	
	RP	69.5±12	72±12	67±12	-
	SFGA	74±9.5	76±9	72±10	-
	InChI-NGN	72.2±1.36	74.4±1.01	68.7±2.45	0.421±0.275

**Table 7:** A summary of the performance on the **DHFR** dataset with results as reported by [1] compared to the InChI-NGN in this work. Missing MCC values in the table reflect missing information in the primary literature.

Design	Method	***Q*** **(%)**	SE(%)	SP(%)	MCC
40% Test Set	SIMCA	75.5	74	71	-
	RP	65	57	73	-
	SFGA	68.5	71	66	-
	InChI-NGN	73.2	73.1	100.0	0.029

Leave-20%-Out	SIMCA	63.5±9.5	57±10	70±9	-
	RP	61±12	57±12	65±12	-
	SFGA	64.5±10.5	65±11.0	64±10.0	-
	InChI-NGN	74.8±1.63	70.3±2.44	77.5±1.72	0.471±0.035

Blood brain barrier results are shown in Table 8. In it, the performance of the InChI-NGN (which denotes an NGN system using the InChI grammar and language) is shown against that of competitors’. Competing machine learning methods were described by Li et al.. They are, Linear Regression (LR), Linear Discriminate Analysis (LDA), Decision Tree (C4.5 DT), k-Nearest Neighbor (k-NN), Probabilistic Neural Network (PNN), and Support Vector Machine (SVM). A method to improve performance was conducted too. Trials were conducted that have Recursive Feature Elimination (RFE) which pruned away redundant or irrelevant elements of the feature vectors. The NGN does not use feature vectors so it cannot benefit from that design.

**Table 8:** A summary of predictive scores for the **BBB** dataset as presented in the work by [2] followed by the performance of the InChI-NGN. Missing ranges for all values other than the SVM work and our own are due to missing information in the primary literature.

Method	***Q*** **(%)**	SE(%)	SP(%)	MCC
LR	57.1	63.6	42.8	0.063
LDA	46.8	40.0	58.4	−0.067
C4.5 DT	73.8	83.7	54.9	0.398
k-NN	70.8	77.0	58.0	0.348
PNN	70.3	76.2	57.8	0.357
SVM	71.0±4.53	89.9±3.16	64.3±13.07	0.524±0.117

LR RFE	71.0	83.9	46.4	0.321
LDA RFE	71.2	78.2	58.3	0.360
C4.5 DT RFE	74.3	80.3	62.8	0.433
k-NN RFE	77.1	85.5	61.4	0.477
PNN RFE	76.1	84.3	62.1	0.481
SVM RFE	83.7±3.90	88.6±7.01	75.0±12.83	0.645±0.080

InChI-NGN	72.0±2.33	77.6±1.72	59.0±3.91	0.355±0.052

Table 9 describes results for the FXa classification dataset. The NGN performs comparably against the results reported for Molecular Interaction Fields (MIF), Fountain et al., 2005 ; and Molecular Kernels (MK), Mohr et al., 2008.

**Table 9:** A comparison of the work done by [3] and [4] against the InChI-NGN for the **FXa** dataset. Missing values are due to lacking information in the primary literature.

Design	Method	***Q*** **(%)**	SE(%)	SP(%)	MCC
⅔ Train, ⅓ Test	A-MIF	88	-	-	-
	MIF-MIF	84	-	-	-
	MK1	94.5	98.7	89.5	-
	MK2	95.2	98.9	87.7	-
	InChI-NGN	83.8	84.3	82.9	0.657

Leave-20%-Out	InChI-NGN	86.4±2.50	88.5±0.87	82.7±0.05	0.705±0.052

Finally, Tables 10 and 11 show the results for each the ER and AR datasets.

**Table 10:** Summary of the Decision Forest performance [5] against the NGN performance on **ER**.

Design	Method	***Q*** **(%)**	SE(%)	SP(%)	MCC
Leave-10%-Out	Decision Forest	81.9	-	-	-

Leave-20%-Out	SMILES-NGN	69.3±2.28	71.7±2.39	66.0±2.74	0.373±0.047
	InChI-NGN	66.1±1.70	69.5±2.65	61.6±1.33	0.309±0.040

**Table 11:** Summary of the NGN performance on **AR**.

Design	Method	***Q*** **(%)**	SE(%)	SP(%)	MCC
Leave-20%-Out	SMILES-NGN	70.3±0.91	71.8±0.46	37.5±11.4	0.052±0.033
	InChI-NGN	76.3±3.20	80.7±2.86	60.6±7.02	0.380±0.098

We now summarize and discuss the results of the classification experiments. The internal cross validation design demonstrates that the NGN is capable of classifying molecules of different problems with varying difficulty. The best accuracy is achieved with the **FXa** dataset at 86.4% concordance. The FXa dataset is well classified by all of the methods shown. This may be owed to the benzamidine moiety shared by each molecule that Fountaine et al. [3] utilized to initially classify the dataset. The NGN classifies 11% more accurately than methods described by Sutherland et al. [1], for the **DHFR** dataset for internal cross validation and within 2% of the leading method for the designed test sets. NGN classification of the **BZR** and **Cox2** datasets are within 2% and 6% concordance of the leading method respectively. The **BZR**, **Cox2** and **DHFR** designed test sets represent particularly challenging problems for machine learning systems as the test sets were selected for maximum dissimilarity [1]. Finally, the NGN classified the **BBB** dataset within 12% of the leading method. The particular difficulty experienced for the **BBB** dataset may be owed to the greater diversity of the compounds described. The NGN does not directly assess solubility or molecular weight which is particularly useful for this problem.

### NGN in QSAR regression experiments

In this section, we discuss the second suite of experiments run. Regression tasks were run using the NGN. The datasets seen in Table 12 were utilized by Sutherland et al., (2004) or retrieved from the Endocrine Disruptor Knowledge Base (EDKB). These datasets have a range in either the pIC_50_ or pK*_i_* which describes the negative base ten logarithm of the proportion of bounded protein-inhibitor pairs to unbound proteins and inhibitors. The datasets used came from the various sources cited. The sample size is shown as well as a citation for the study of origin of each. Each dataset varies in its representation of low affinity to high affinity binders.

**Table 12:** A summary of the real-valued ranges of activity for datasets used in regression experiments.

Dataset	Dataset Full Name	Size	Range	Units	Reference
ACE	Angiotensin Converting Enzyme	114	[2.1, 9.9]	pIC_50_	[6][26]
AChE	Acetylcholinesterase	111	[4.3, 9.5]	pIC_50_	[6]
AR	Androgen Receptor	146	[−3.56, 2.27]	logRBA	[18]
BZR	Benzodiazepine Receptor	163	[5.5, 8.9]	pIC_50_	[6]
Cox2	Cyclooxygenase-2	282	[4.0, 9.0]	pIC_50_	[6]
DHFR	Dihydrofolate Reductase	397	[3.3, 9.8]	pIC_50_	[6]
ER	Estrogen Receptor	131	[−4.50, 2.60]	logRBA	[16][17]
GPB	Glycogen Phosphorylase B	66	[1.3, 6.8]	pK*_i_*	[6][27]
Therm	Thermolysin	76	[0.5, 10.2]	pK*_i_*	[6][26]
Thr	Thrombin	88	[4.4, 8.5]	pK*_i_*	[6][28]

In order to evaluate the performance of the NGN in regression tasks, the following quantities are calculated in order to characterize how much deviation there is from the actual output to the target output. The two quantities, predictive residual sum of squares (PRESS) and standard deviation (SD), are intermediate values used to calculate a value commonly used in the literature,  to characterize this deviation. One can consider the  score to be PRESS normalized against SD. Note that SD is calculated with respect to the test set only.

Table 13 details the results for the internal cross validation, Leave-5%-Out. In it, we provide the  score followed by a standard deviation after the plus minus symbol (±). Twenty trials total were run on each dataset, once each for each Leave-5%-Out test group.

**Table 13:** The  scores for converging Leave-5%-Out Cross Validation regression experiments.

Grammar	Dataset	Dataset Full Name	
SMILES	**ACE**	Angiotensin Converting Enzyme	0.386±29.20
	**AR**	Androgen Receptor	0.382±18.29
	**ER**	Estrogen Receptor	0.349±21.82
	**GPB**	Glycogen Phosphorylase B	0.253±43.18
	**AChE**	Acetylcholinesterase	0.193±35.26
	**Therm**	Thermolysin	−0.288±210.30

InChI	**ER**	Estrogen Receptor	0.476±11.16
	**ACE**	Angiotensin Converting Enzyme	0.383±15.09
	**Cox2**	Cyclooxygenase-2	0.279±8.97
	**Therm**	Thermolysin	0.247±25.96
	**AR**	Androgen Receptor	0.119±79.50
	**Thr**	Thrombin	0.088±99.79
	**AChE**	Acetylcholinesterase	0.061±39.70
	**GPB**	Glycogen Phosphorylase B	−0.180±104.87

Table 14 shows how our method compared against other published methods. We report standard deviations after the plus minus sign (±). Where a value is not reported in previous work, it is left blank. The regression results indicate that the NGN performed better in  with both a higher average score and narrower standard deviation for the designed test sets than compared with the Leave-5%-Out Cross Validation. A narrower spread indicates greater model stability. This is a desirable trait as it provides a quantitative basis for reliability. The better performance achieved in the designed test set is surprising as eight of the ten designed datasets were selected given a maximum dissimilarity algorithm [6]. The NGN method performed better than all other methods for the **GPB**, **ACE**, **AChE**, **Cox2**, **Thr** and **DHFR** datasets. The least desirable performance was obtained on the **BZR** dataset where a negative score is indicated implying that the system only captured noise. The **BZR** dataset also corresponds to near bottom performance for the remaining methods as well indicating that it is particularly difficult. The InChI-NGN method achieved its best results with the **ACE** dataset which is also the second best performance of the SMILES-NGN. It is possible that the salient physical properties for this dataset are particularly well exposed in the SMILES and InChI strings.

**Table 14:** A summary of  scores for the NGN compared to other methods on datasets described for regression in this work.

Dataset	SMILES-NGN	InChI-NGN	CoMFA	EVA	HQSAR	2.5D	MK1	MK2
GPB	0.79±0.23	0.48±2.90	0.42	0.49	0.58	0.04	—	—
ACE	0.74±0.46	0.78±0.31	0.49	0.36	0.30	0.51	0.58	0.55
AChE	0.68±0.80	0.60±0.78	0.47	0.28	0.37	0.16	0.50	0.48
Cox2	0.56±1.33	0.37±4.28	0.29	0.17	0.27	0.27	—	—
Therm	0.47±2.72	0.52±1.59	0.54	0.36	0.53	0.07	—	—
BZR	−0.29±17.30	0.11±8.74	0.00	0.16	0.17	0.20	0.34	0.36
Thr	—	0.70±0.72	0.63	0.11	−0.25	0.28	—	—
DHFR	—	0.66±0.94	0.59	0.57	0.63	0.49	0.64	0.65

### SMILES-NGN toxicity prediction

#### *Experimental method*

To develop a list of toxins, the Carcinogenic Potency Database (CPDB) was examined [20]. This list was then used to look up the structures of the identified toxins by acquiring Structure Data Files (SDF) and Information Systems (MDL) Molfiles [21] from various sources. Next, the lethal dose for 50 percent of the population (LD50) was found for each molecule. The LD50 is representative of a substance’s toxicity for a given population because it avoids the obscurity of lower or higher doses in the population. The toxin names, molecular structures, target organs, organisms of study, and LD50 values were incorporated into a new database, which we provide for the community for an earlier publication with some preliminary results showing a proof-of-concept model [19] (Refer to *Additional file *2 for toxicity dataset description). In this publication, we extend this study with a statistical analysis using the Wilcoxin test. We remark that this experiment does not claim to link carcinogenicity and lethality; rather, we used a list of toxins that happened to be carcinogenic as the basis of our dataset. All predictions made, relate to toxicity (i.e. LD50 death rate) from all causes, not simply cancer alone.

In the database, there were 173 unique toxicants with some of these compounds affecting multiple organs (thereby increasing the amount to 248 data points). Oral LD50 values in this experiment ranged from 0.48 to 20,000 mg/kg. All compounds are considered toxic at a significantly large dosages. Therefore, no inactive or non-toxic molecules were used within the range of this experiment. The database was then separated into subsets according to species and organ.

The Chemistry Development Kit (CDK) [22] was used to produce QSAR or traditional molecular descriptors. OpenBabel [23] was used to generate the SMILES representation, given SDF and MDL files for the NGN. A normalization of the LD50 data was performed to scale the data into the range of outputs produced by ANN and NGN. By using the following formula, the known LD50s for each data set were normalized into the range of 0.2 to 0.8 before insertion into either ANN or NGN.

Here, ‘val’ is the current value, ‘min’ is the minimum value of the data set, and ‘max’ is the maximum value of the data set. Three datasets were created; one of which is an accumulation of all of the data, one a subset of animals (mouse and rat), and the final one being a subset of animal organ combinations (for example, rat kidney or mouse liver) . Each dataset was used to test for the convergence competence of both neural networks. A preliminary convergence test was designed to evaluate the system’s ability to learn the complete dataset. This preliminary convergence test was required to ensure that the datasets contain few enough contradictions and ambiguities that the neural network is capable of producing worthwhile predictions. Convergence is defined as the root mean squared error (RMSE) being less than a threshold. The system parameters that we have derived experimentally based on these convergence tests for the comparison of the ANN and NGN are shown in Table 15. Neither the dataset, being an accumulation of all of the data, nor the animal subset passed the convergence test for either ANN or NGN. The animal organ combination dataset produced more desirable results, though three datasets were found to be problematic; (i) the rat-liver (which could not be learned by the ANN), (ii) the mouse-liver (which could not be learned by either the ANN or the NGN), (iii) the mouse-kidney (which could not be learned with the grouped dataset by either the ANN or the NGN—see below). All successful datasets, those of which passed the convergence test, were applied to the appropriate methods. Training and prediction occurred given two means of breaking apart the data: grouped and random. Both methods were based on a five-fold cross-validation. The grouped method separated the data apart into five equally-sized clusters which were evenly distributed across the range of outputs, such that four clusters were used in training and one cluster was used in prediction. These groups were then used repeatedly for 100 tests in the case of the ANN and 50 tests in the case of the NGN due to the processing time required for this method. The random method randomly selected out one fifth to be used for prediction for each separate test. Thus, the random test produces more variability. The difference in grouping method allowed for a comparison of variability between the molecules introduced by the system, where random training introduced more variability than group.

**Table 15:** Description of the neural networks’ parameters used for the experiment

									RMSE Threshold	
**Neural Net**	**Number of Layers Node**	**Number of Input Nodes**	**Number of Hidden Nodes**	**Number of Output**	***η***	***α***	**Weight Values Range**	**Epoch**	**Convergence**	**Training**

ANN	3	Same as feature vector	2	1	0.9	0.3	(−0.3,0.3)	50,000	0.02	0.05
NGN	Dynamic	Dependant on input token	12	1	0.3	0.3	(−1.6,−1.0) or (1.0,1.6)	10,000	0.03	0.05

*activation value of 1.0

*hidden nodes were tested for best performance, leading to 12 hidden nodes to be used with the NGN for the toxicity dataset

To provide validity to the system estimates, a statistical analysis was performed. The difference between the target normalized LD50 score and the actual output given by the neural net was taken and summed for each molecule processed in a given trial. This number is defined as Epsilon. The standard deviation of the difference for each prediction and actual output pair were also taken. As Epsilon approaches zero this is an indication that there are smaller residuals against the target data, portraying a more accurate model (ie. good on average). For a model to be precise (i.e. stable), the standard deviation of the Epsilon value must approach zero as well. This statistical analysis was performed for both NGN and ANN estimates. A Wilcoxon signed-rank test, on Epsilon and standard deviation for each trial, was used to determine which method performed better. This test is a significant improvement over the analyses done in some of the preliminary publications. Correlation coefficients (CC) were calculated for the NGN and ANN trials to provide a quality of least squares fitting between the known LD50 values and the neural net estimates. The CC’s were calculated using:

Here, ‘x’ represented the known LD50 values and ‘y’ represented the neural net estimates.

The results achieved from this experiment show that the SMILES-NGN provides better overall estimates of toxicity when compared against ANNs processing traditional descriptors in QSAR problems. The SMILES-NGN produced results closer to the correct response on average (Epsilon is closer to zero), and also produced more stable results (standard deviation is lower). On average, the SMILES-NGN outperformed the QSAR-ANN with lower Epsilon and standard deviation. The SMILES-NGN was able to converge on the rat liver data set while the ANN could not. From the results of the Wilcoxon signed-ranked test, which were applied only to the datasets where the ANN approach could generate a result, we deduced that random training provided a better estimate of LD50’s compared to grouped training method design. It is within 95% confidence that the SMILES-NGN performed better than ANNs with descriptors. This conclusion cannot be drawn for the group training data. Even though it may be true that the SMILES-NGN results are below those of the ANN for group training, it is not within 95% confidence (only results of best descriptors are shown). It is significant that the best descriptor is not consistent across datasets, while the exact same NGN was used for all experiments.

From this experiment, it has been identified that the SMILES-NGN was the better candidate when compared with ANNs using traditional descriptors. This conclusion is based on two criteria:

(i) No domain-specific knowledge is required other than the structural interpretation embedded in the SMILES grammar by its designers. Even with the toxicant dataset developed here, the QSAR descriptors must be designed by experts and are highly specific.

(ii) With SMILES the entire molecule is submitted to the neural network, not just a relational representation (QSAR).

As a point of comparison, we implemented ANN and compared NGN results. We believe that to do a proper comparison and justice to other techniques, such as support vector machines or KNN, it would be better for users more experienced in those techniques to apply them to the dataset. Therefore, we are making the dataset available and welcome others to try to produce better results with their preferred methods.

#### *Experimental results*

**Figure 5:**
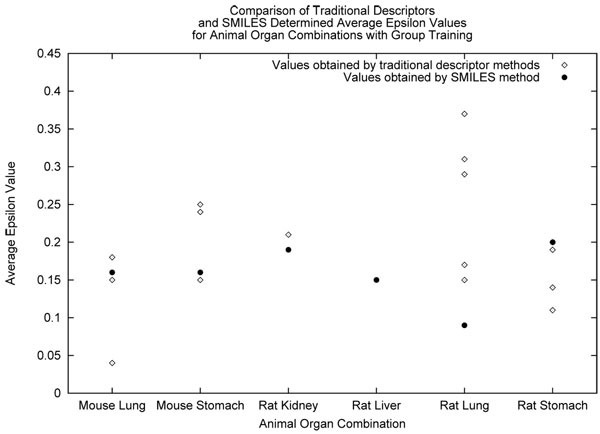
Comparison of group method determined epsilon values.

**Figure 6:**
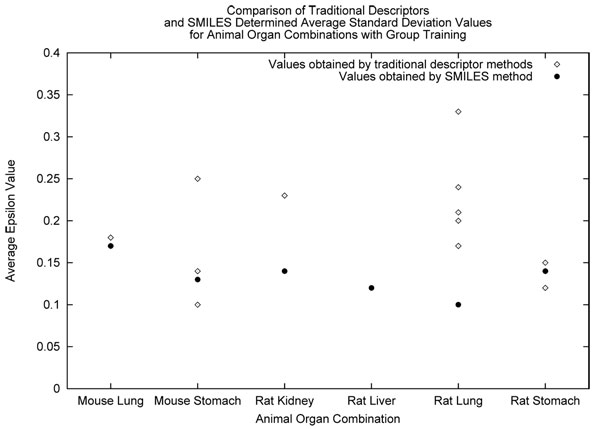
Comparison of group method determined standard deviation values.

**Figure 7:**
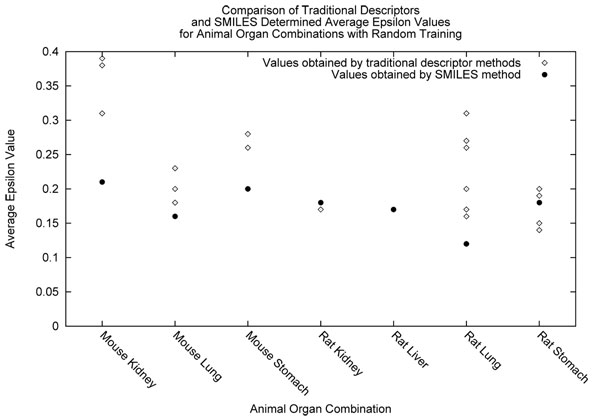
Comparison of random method determined epsilon values.

**Figure 8:**
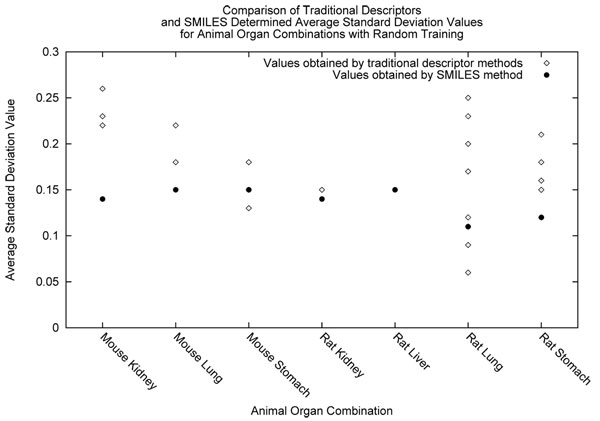
Comparison of random method determined standard deviation values.

We observed the successful descriptors for each dataset under certain training conditions (grouped or random) and training statistic (epsilon or standard deviation). We examined the average performance of each individual feature vector or as well as our approach using SMILES. The same assumptions for epsilon and standard deviation can be made, where the closer the statistic is to zero the better it has performed. We show the average epsilon values acquired for the SMILES-NGN and ANN methods in Figure 5. In this graph, each of the descriptors tried with the ANN are shown as their own datapoint above each animal and organ combination. In Figure 6, we describe the same suite of experiments as before but the average standard deviation is shown instead. The above two figures mentioned summarize the results for the grouped treatment of data. We summarize the results for the random treatment of data in Figures 7 and 8 for each epsilon and standard deviation respectively. Most often our method with SMILES scored lowest, demonstrating that it outperformed ANN descriptors. Finally, When NGN and ANN feature vectors for animal organ datasets were compared: (i) In the case of random training conditions, NGN outperformed ANN for epsilon and standard deviation 5 out of 7 datasets (ii) In the case of grouped training condition, NGN outperformed ANN for epsilon 3 out of 6 datasets and for standard devation 4 out of 6 datasets These results show that more than half of all results were better estimates with NGN then ANN feature vectors. It is significant that the range for NGN estimates are a quarter or a half that of ANN’s depending on animal organ dataset. This result is significant because the lower the range for a statistic, the better the model stability, leading to more reliable results. Refer to *Additional file *1 for graphs, complete data and statistical analysis of the results.

## Conclusions

We conclude that our method is superior to those which have been previously examined based on three points. First, our method does not require the expert knowledge that is necessary to design the feature vectors or kernel matrices used by other methods. We found our approach, of using the SMILES and InChI grammars as the sole source of chemistry-specific prior knowledge, worked exceptionally well when compared to more problem-specific approaches as evidenced by the performance of our system across a very broad range of problem domains. We are hopeful that this trend will continue across other descriptor languages. We believe that one reason for this success is the second advantage of our system.

This advantage being that it creates a lossless representation of molecules for presentation to the learning system. Approaches that reduce complex molecular structures to a small number of numerical quantities or kernel matrices remove data from the problem before the learning phase. In an ideal case the lost data would be irrelevant to the problem under consideration, but in practice this may not be the case. Thus, experts are required to judiciously design representations that do not lose any problem-relevant information. By contrast, our approach is not lossy, so the learning component in the system can adapt to use all structural information.

The third advantage of our system is that it is able to leverage the work done to formulate chemical structure description languages, like SMILES and InChI. These languages have been designed to represent three dimensional structural information in a most useful way. The success of our system is a testament to the effectiveness of these representative systems.

The NGN has two unique challenges not faced by competitor methods. These challenges contribute to weaker performances observed. First, there is the well-known problem of signal decay down a recurrent neural network stack. This problem corresponds to weaker confidence when working on larger molecules as the error propagation needed for training has a larger number of transfer steps to travel between the output node of the NGN and the deepest leaves that map the right most tokens of the SMILES or InChI string. Second, the NGN architecture is highly specialized at learning the topology of an input sequence, but not the sequence itself. That is, the NGN cannot interpret the distance between two tokens, nor the length of a particular repeating stretch of symbols. It is instead designed to recognize the occurrence of a token in the local context of nearby tokens and internal activation layers.

Future work will look at: 1) Expanding our studies to even more problems from the domain of cheminformatics. 2) Comparing to even more alternative approaches. 3) Investigation of other grammatical representations beyond SMILES and InChI. 4) Moving beyond chemistry to apply NGNs to other problem domains where formal grammars have been developed to represent objects of interest.

## Authors’ contributions

Stefan Kremer served as M.Sc. supervisor and undergraduate project supervisor to Eddie Ma and Christopher Cameron respectively. He guided the objectives of the research, analysis methods, etc.. Eddie Ma developed the software to implement the NGN and conducted the QSAR experiments and analysis. Christopher Cameron designed, conducted and analysed the toxicology experiments. Each author wrote first drafts of the sections corresponding to the work that they conducted and then worked as a team on the crafting of the complete document.

## Competing interests

All authors declare that there are no competing interests, financial or non-financial, and therefore have nothing to declare.

## Supplementary Material

Additional file 2 We have included the following item in an additional PDF file named additional_file_2.pdf. We have provided a description of the SMILES-NGN dataset which includes all molecules and LD50’s with their respective sources. Click here for file

Additional file 1 We have included the following items in an additional PDF file named additional_file_1.pdf. First, the performance of the NGN in classification tasks is visualized with a suite of graphs. For each dataset on which the NGN achieved convergence, a graph is rendered showing the NGN’s classification concordance given a domain of classification threshold values. Second, additional experimental performance data from the toxicology task are rendered in tables describing (1) the epsilon value, standard deviation and correlation coefficient for each the ANN and NGN; (2) the difference in performance between the ANN and NGN using the Wilcoxon statistical test; and (3) a summary of the best performing system for each pair of matched ANN and NGN trials. Click here for file
