# Supplementary material for: Classifying and scoring of molecules with the NGN: new datasets, significance tests, and generalization
Source: BMC Bioinformatics. 2010 Oct 26;11(Suppl 8):S4. doi: 10.1186/1471-2105-11-S8-S4 (PMC2966291; doi:10.1186/1471-2105-11-S8-S4)
Supplement: Additional file 2 — We have included the following item in an additional PDF file named additional_file_2.pdf. We have provided a description of the SMILES-NGN dataset which includes all molecules and LD50’s with their respective sources. [file 1471-2105-11-S8-S4-S2.pdf]

A Benchmark Molecular Data Set for QSAR and SMILES Descriptors

By Chris Cameron, 12/04/2009

Below, all the molecules that are listed were used within the experiment (Table-1). Comparison between the source number (either oral LD50 or SDF/Molfile), and the accompanying table (Table-3 and Table-4, respectively) will show what source was used to obtain the value or file. For example:

Figure-1: Example molecule table for the explanation of how to determine sources of obtained values for oral LD50 and SDF/Molfile

Organ	Species	No.	Molecule	SDF/Molfile Source	Oral LD50 (mg/kg)	Oral LD50 Source	2D/3D
**LIVER**	**RAT**	3	Aflatoxin B1	21	4800	3	3D

'Aflatoxin B1' which affects the rat's liver has an Oral LD50 source of 1, from Table-3, the source was 'http://msds.chem.ox.ac.uk/'. The SDF/Molfile source is number 3 and the corresponding source from Table-4 is 'http://www.chemexper.com/'.

Table-1: Summary of molecules with oral LD50's and SDF/Molfile dimension relating to organ and animal to which carcinogenic activity was examined, with source numbers from which they were obtained

Organ	Species	No.	Molecule	SDF/Molfile Source	Oral LD50 (mg/kg)	Oral LD50 Source	SDF/Molfile Dimension (2D/3D)
**LIVER**	**RAT**	1	Acetamide	21	7000	1	3D
		2	Acetaminophen	21	1944	2	3D
		3	Aflatoxin B1	21	4800	3	3D
		4	Benzidine	21	309	1	3D
		5	Budesonide	21	3200	5	3D
		6	Captafol	21	6200	6	3D
		7	Carbon tetrachloride	21	2350	1	3D
		8	Chlorendic acid	21	1770	7	3D
		9	Chlorobenzene	21	1110	1	3D
		10	Chloroform	21	1194	1	3D

		11	Clofibrate	21	940	8	3D
		12	Coumarin	21	293	3	3D
		13	p-Cresidine	21	1450	9	3D
		14	Crotonaldehyde	21	206	1	3D
		15	Cupferron	21	199	1	2D
		16	DDT	21	87	1	3D
		17	Dichloroacetic acid	21	2820	4	3D
		18	Diethylstilbestrol	21	3000	1	3D
		19	2,4-Dinitrotoluene	22	268	1	2D
		20	Dinitrotoluene	21	790	9	3D
		21	1,4-Dioxane	22	5.2	10	2D
		22	Dipentylnitrosamine	21	2500	1	3D
		23	Doxylamine succinate	21	600	1	2D
		24	Ethinyl estradiol	21	2952	1	3D
		25	Ethyl alcohol	21	7060	1	3D
		26	Ethylene thiourea	21	1832	9	3D
		27	Fluconazole	21	1271	5	3D
		28	Furan	21	890	11	3D
		29	Furfural	21	65	4	3D
		30	Hydrazine	21	60	1	3D
		31	Hydrazine sulfate	21	601	1	2D
		32	Isoniazid	21	160	5	3D
		33	Kepone	21	91	1	3D
		34	Methyleugenol	21	1179	1	3D

		35	Metronidazole	21	3000	5	3D
		36	Sodium Nitrite	21	180	4	2D
		37	Nitrobenzene	21	349	4	3D
		38	Pyrilamine maleate	21	36	1	2D
		39	Safrole	21	1950	1	3D
		40	Selenium sulfide	23	139	5	2D
		41	Sterigmatocystin	21	120	12	3D
		42	Tamoxifen citrate	21	1190	5	2D
		43	Thioacetamide	23	301	5	3D
		44	Triamcinolone acetonide	21	500	13	2D
		45	Vinyl acetate	21	2920	1	3D
		46	Vinyl bromide	21	500	1	3D
		47	Vinyl chloride	21	500	1	3D
	MOUSE	48	Auramine-O	21	480	4	2D
		49	Coumarin	21	196	3	3D
		50	Doxylamine succinate	21	470	1	2D
		51	Aldrin	21	44	14	3D
		52	4-Aminodiphenyl	25	205	1	2D
		53	Benzidine	21	214	20	3D
		54	Benzyl acetate	21	830	17	3D
		55	Bis-2-chloroethylether	21	209	1	3D
		56	Bromodichloromethane	21	2570	17	3D
		57	1,3-Butadiene	26	3210	1	3D
		58	Butylated hydroxytoluene	21	650	5	3D

		59	Carbon tetrachloride	21	8623	1	3D
		60	Chloral hydrate	21	1100	17	3D
		61	Chloramben	21	3725	17	3D
		62	1-Chloro-2-nitrobenzene	23	135	1	2D
		63	1-Chloro-4-nitrobenzene	21	650	1	3D
		64	Chlorobenzilate	21	729	1	3D
		65	Chlorodibromomethane	21	800	17	3D
		66	Sodium cyclamate	23	1700	5	2D
		67	DDT	21	135	1	3D
		68	1,4-Dichlorobenzene	21	2950	1	3D
		69	1,2-Dichloroethane	21	413	17	3D
		70	1,2-Dichloropropane	23	860	1	2D
		71	Dicofol	21	420	1	3D
		72	5,5-Diphenylhydantoin	21	150	17	3D
		73	Doxylamine succinate	21	470	1	2D
		74	2-Ethylhexanol	21	3350	3	3D
		75	Furfural	21	400	4	3D
		76	Gentian violet	21	96	17	2D
		77	Glycidol	21	431	1	3D
		78	Griseofulvin	21	50	17	3D
		79	Hexachlorobenzene	21	4	7	3D
		80	Hydrazine sulfate	21	740	1	3D
		81	Hydroquinone	21	350	17	3D
		82	Methyl tert-butyl ether	21	5960	5	3D

		83	Methylhydrazine	21	57	1	3D
		84	2-Methylimidazole	21	1400	3	3D
		85	5-Nitro-o-anisidine	21	1060	1	3D
		86	o-Nitroanisole	21	1300	1	3D
		87	Nitrobenzene	21	590	5	3D
		88	6-Nitrobenzimidazole	21	125	5	3D
		89	Nitrofen	21	450	1	3D
		90	Nitromethane	21	950	1	3D
		91	o-Nitrotoluene	21	970	1	3D
		92	Ochratoxin A	21	46	17	3D
		93	Pentachloronitrobenzene	21	1400	1	3D
		94	Phenobarbital	21	168	1	3D
		95	Phenylbutazone	21	270	1	3D
		96	Piperonyl butoxide	21	3800	1	3D
		97	Probenecid	21	1666	1	3D
		98	Safrole	21	2350	17	3D
		99	Selenium sulfide	21	370	17	3D
		100	1,1,1,2-Tetrachloroethane	221	780	1	2D
		101	Toxaphene	21	112	1	2D
		102	Tributyl phosphate	21	1189	17	3D
		103	2,4,6-Trichloroaniline	21	5800	1	3D
		104	1,1,2-Trichloroethane	21	378	1	3D
		105	2,4,6-Trichlorophenol	23	770	1	2D
		106	1,2,3-Trichloropropane	23	369	17	2D

		107	Tris(2-chloroethyl)phosphate	23	1866	1	2D
		108	Tris(2-ethylhexyl)phosphate	23	8000	1	2D
		109	Urethane	21	2500	1	3D
**STOMACH**	**RAT**	110	Acrylonitrile	21	78	1	3D
		111	Benzene	21	930	1	3D
		112	Butylated hydroxyanisole	21	2000	1	3D
		113	Catechol	21	260	1	3D
		114	3-Chloro-2-methylpropene	23	848	3	3D
		115	Cupferron	21	199	1	2D
		116	1,2-Dibromoethane	21	108	1	3D
		117	1,2-Dichloroethane	21	500	4	3D
		118	Diglycidyl resorcinol ether	21	2570	1	3D
		119	Dimethyl hydrogen phosphite	21	3040	3	3D
		120	Epichlorohydrin	21	90	1	3D
		121	Ethyl acrylate	21	800	1	3D
		122	Ethylene oxide	21	72	1	3D
		123	Glycidol	21	420	1	3D
		124	2,4-Hexadienal	21	300	1	3D
		125	Mercuric chloride	21	1	1	2D
		126	4-Methoxyphenol	21	1600	1	3D
		127	Methyleugenol	21	1179	1	3D
		128	2-Nitrofluorene	21	3400	1	3D
		129	Omeprazole	21	2210	1	3D
		130	Phenacetin	21	3600	1	3D

		131	Pivalolactone	21	1470	1	3D
		132	1,2-Propylene oxide	22	380	1	2D
		133	Styrene oxide	21	2000	1	3D
		134	Sulfallate	21	850	1	3D
		135	Telone II	21	300	15	3D
	**MOUSE**	136	Acrylonitrile	21	27	1	3D
		137	Benzaldehyde	21	2020	17	3D
		138	Benzotrichloride	21	702	1	3D
		139	Benzyl acetate	21	830	17	3D
		140	Benzyl chloride	21	1500	17	3D
		141	2-Butoxyethanol	21	1230	17	3D
		142	1,3-Butadiene	21	3210	1	3D
		143	Butylated hydroxyanisole	21	1100	1	2D
		144	Catechol	21	260	1	3D
		145	3-Chloro-2-methylpropene	21	1370	17	3D
		146	1,2-Dibromoethane	21	3100	17	3D
		147	Ethyl acrylate	21	1799	1	3D
		148	Glycidol	21	431	1	3D
		149	Styrene oxide	21	1500	1	3D
		150	Tris(2-chloroethyl)phosphate	21	1866	1	3D
		151	Vinyl acetate	21	1600	17	3D
**KIDNEY**	**RAT**	152	Aflatoxin B1	21	0.48	1	3D
		153	1-Amino-2,4-dibromoanthraquinone	24	20000	9	2D
		154	2-Amino-4-nitrophenol	21	2400	16	3D

		155	Potassium Bromate	21	321	4	2D
		156	Sodium Barbital	21	600	3	2D
		157	Bromodichloromethane	21	430	17	3D
		158	tert-Butyl alcohol	21	3500	1	3D
		159	Cadmium Chloride	21	88	1	2D
		160	Captafol	21	6200	18	3D
		161	Captan	21	9000	1	3D
		162	Chloroform	21	1194	1	3D
		163	Chlorothalonil	21	28.2	19	3D
		164	Cinnamyl anthranilate	21	5000	1	3D
		165	Coumarin	21	293	3	3D
		166	1,4-Dichlorobenzene	23	500	1	3D
		167	Dimethyl methylphosphonate	21	8210	5	3D
		168	Ethylbenzene	21	3500	5	3D
		169	Glycine	21	7930	1	3D
		170	Hexachlorobutadiene	21	270	1	2D
		171	Hexachloroethane	21	6000	1	3D
		172	Hydroquinone	21	320	1	3D
		173	Isophorone	21	2330	1	3D
		174	Isoprene	21	5240	5	3D
		175	Lead acetate	21	4665	4	2D
		176	d-Limonene	21	4400	1	3D
		177	Methyl tert-butyl ether	21	4000	4	3D
		178	Methyleugenol	21	1179	1	3D

		179	Nitrilotriacetic acid	21	1100	4	3D
		180	o-Nitroanisole	21	740	1	3D
		181	Nitrobenzene	21	780	5	3D
		182	2-Nitrofluorene	21	3400	1	3D
		183	1-[(5-Nitrofurfurylidene)amino]hydantoin	21	604	1	3D
		184	N-Nitrosodiethylamine	21	280	1	3D
		185	N-Nitrosodimethylamine	21	58	1	3D
		186	Ochratoxin A	21	20	17	3D
		187	Phenacetin	21	3600	4	3D
		188	Phenazone	21	1705	17	3D
		189	Phenylbutazone	21	245	5	3D
		190	Pyridine	21	891	1	3D
		191	Tetrachloroethylene	21	2629	5	3D
		192	Tetrahydrofuran	21	2816	1	3D
		193	Tris(2-chloroethyl)phosphate	21	1230	1	3D
		194	Tris-(1,3-dichloro-2-propyl)phosphate	25	1850	1	2D
		195	Vinyl chloride	21	500	1	3D
	**MOUSE**	196	Potassium Bromate	21	3120	17	2D
		197	Bromodichloromethane	21	2570	17	3D
		198	1,3-Butadiene	21	3210	1	3D
		199	2,4-Dinitrotoluene	21	268	17	3D
		200	Furfuryl alcohol	21	160	5	3D
		201	Hydroquinone	21	245	17	3D
		202	Tris(2-chloroethyl)phosphate	21	1866	1	2D

		203	Vinylidene chloride	21	194	17	3D
LUNG	RAT	204	Sodium Dichromate	21	50	4	2D
		205	Bromoethane	21	1350	3	2D
		206	Cadmium chloride	21	60	1	2D
		207	Cadmium sulphate	21	280	4	3D
		208	2,2-Bis(bromomethyl)-1,3-propanediol	21	2000	9	3D
		209	Dimethyl hydrogen phosphite	21	8210	17	3D
		210	1,2-Epoxybutane	21	500	1	3D
		211	Hydrazine	21	60	1	3D
		212	Hydrazine sulfate	21	601	1	2D
		213	Isobutyl nitrite	21	410	1	3D
		214	N-Nitrosodimethylamine	21	58	1	3D
		215	o-Nitrotoluene	21	891	1	3D
		216	Tetranitromethane	21	130	1	3D
	MOUSE	217	5-Azacytidine	21	572	17	3D
		218	Benzene	21	4700	1	3D
		219	Benzotrichloride	21	702	1	3D
		220	1,3-butadiene	27	3210	1	3D
		221	Butylated hydroxytoluene	21	650	5	3D
		222	Chlorambucil	21	80	1	3D
		223	Cobalt sulfate heptahydrate	21	584	17	2D
		224	Coumarin	21	293	3	3D
		225	Cyclophosphamide	21	137	1	3D
		226	Dacarbazine	21	350	2	3D

		227	DDT	21	135	1	3D
		228	1,2-Dichloroethane	21	413	17	3D
		229	5-Fluorouracil	21	115	1	3D
		230	Glycidol	21	431	1	3D
		231	Hydrazine	21	59	17	3D
		232	Hydrazine sulfate	21	740	1	2D
		233	Isobutyl nitrite	21	205	1	3D
		234	Isoniazid	21	176	7	3D
		235	Methyl methanesulfonate	21	290	1	3D
		236	Methylene chloride	21	873	3	3D
		237	Methylhydrazine	21	57	1	3D
		238	Metronidazole	21	3800	1	3D
		239	Molybdenum trioxide	21	127	17	2D
		240	Naphthalene	21	533	1	3D
		241	Nitrobenzene	21	590	5	3D
		242	Nitromethane	21	950	1	3D
		243	Selenium sulfide	21	370	17	2D
		244	Styrene	21	316	1	3D
		245	Tetranitromethane	21	375	1	3D
		246	Urethane	21	2500	1	3D
		247	Vinylidene chloride	21	194	5	3D

NOTE: Certain molecules were obtained with LD50 values with ranges, due to the inability of the neural net or neural grammar network to handle the range, the largest value in the range was recorded (due to this study examining lethality)

Table-2: List of obtained L D50's that were altered for the study

Organ	Species	No.	Molecule	Oral LD50 Source	Original Oral LD50 (mg/kg)	Used Oral LD50 (mg/kg)
LIVER	RAT	248	Diethylstilbestrol	21	>3000	3000
		249	Triamcinolone acetonide	21	>500	500
	MOUSE	250	Bromodichloromethane	21	450 and 2570	2570
		251	Griseofulvin	21	equal or >50	50
		252	Hydroquinone	21	245 and 350	350
STOMACH	RAT	253	3-Chloro-2-methylpropene	23	848 and 580	848
	MOUSE	254	Benzaldehyde	21	28 and 2020	2020
		255	2-Butoxyethanol	21	1167 and 1230	1230
KIDNEY	RAT	256	Aflatoxin B1	21	0.4 and 0.48	0.48
		257	1-Amino-2,4-dibromoanthraquinone	24	>20000	20000
		258	Chlorothalonil	21	>28.2	28.2
	MOUSE	259	Potassium Bromate	21	289 and 3120	3120
		260	Bromodichloromethane	21	450 and 2570	2570
		261	2,4-Dinitrotoluene	21	177 and 268	268
LUNG	RAT	262	2,2-Bis(bromomethyl)-1,3-propanediol	21	>2000	2000
		263	Dimethyl hydrogen phosphite	21	840-8210	8210

Table-3: List of sources from which the oral LD50's were obtained from

Source Number	Site From which LD50 was Obtained From
1	http://msds.chem.ox.ac.uk/
2	http://www.drugbank.ca/
3	https://fscimage.fishersci.com/
4	http://www.jtbaker.com/
5	http://www.sciencelab.com/
6	http://pubchem.ncbi.nlm.nih.gov/
7	http://www.inchem.org/
8	http://www.lifescience-online.com/
9	http://www.chemicalland21.com/
10	http://www.epa.gov/
11	http://www.coleparmer.com/
12	http://fermentek.co.il/
13	http://www.paddocklabs.com/
14	http://www.pesticideinfo.org/
15	http://www.dowargo.com/
16	http://www.analytyka.com.mx/
17	http://www.21cnlab.com/
18	http://www.osha.gov/
19	http://pmep.cce.cornell.edu/
20	http://www.intox.org/

Table-4: List of sources from which SDF/Molfile were obtained

Source Number	Site from which SDF/Molfile was Obtained
21	http://pubchem.ncbi.nlm.nih.gov/
22	http://www.ebi.ac.uk/
23	http://www.chemexper.com/
24	http://molecules.gnu-darwin.org/
25	http://www.chemicalbook.com/
26	http://www.dfmg.com.tw/
27	http://webbook.nist.gov/
