# Supplementary material for: Classifying and scoring of molecules with the NGN: new datasets, significance tests, and generalization
Source: BMC Bioinformatics. 2010 Oct 26;11(Suppl 8):S4. doi: 10.1186/1471-2105-11-S8-S4 (PMC2966291; doi:10.1186/1471-2105-11-S8-S4)
Supplement: Additional file 1 — We have included the following items in an additional PDF file named additional_file_1.pdf. First, the performance of the NGN in classification tasks is visualized with a suite of graphs. For each dataset on which the NGN achieved convergence, a graph is rendered showing the NGN’s classification concordance given a domain of classification threshold values. Second, additional experimental performance data from the toxicology task are rendered in tables describing (1) the epsilon value, standard deviation and correlation coefficient for each the ANN and NGN; (2) the difference in performance between the ANN and NGN using the Wilcoxon statistical test; and (3) a summary of the best performing system for each pair of matched ANN and NGN trials. [file 1471-2105-11-S8-S4-S1.pdf]

Classifying and scoring of molecules with the NGN: new datasets, significance tests, and generalization

Eddie Y.T. Ma[1] , Christopher J.F. Cameron[2] , Stefan C. Kremer*[2]

[1] Department of Biology at the University of Waterloo, Waterloo, Ontario, Canada
[2] School of Computer Science at the University of Guelph, Guelph, Ontario, Canada

Email: Eddie Y.T. Ma - e3ma@uwaterloo.ca; Christopher J.F. Cameron - ccameron@uoguelph.ca; Stefan C. Kremer*-skremer@uoguelph.ca;

*Corresponding author

Supplementary materials

Cheminformatics classification graphs

The nine graphs, Figures 1 – 4 describe grammars and datasets that converged during training. In these graphs, every single response of the graph is sorted from lowest to highest. Each response value is in turn used as the threshold value. The range of the graph is the number of correct predictions given a value as the threshold. A bulge upward indicates a system that performs favourably; a straight line indicates random guessing; and a bulge downward indicates a poorly performing system. The bounding parallelogram indicates the possible range of answers.

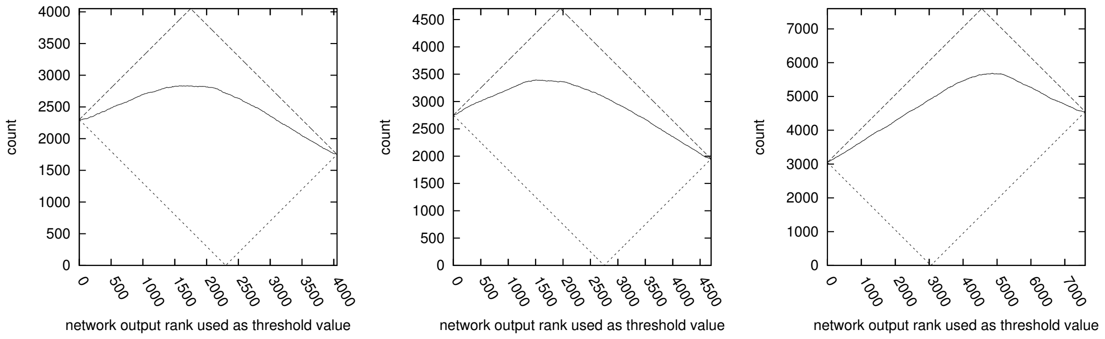

(a) BZR 20% CV (b) Cox2 20% CV (c) DHFR 20% CV

Figure 1: A summary of performance on three classification cross validation experiments, InChI-NGN on benzodiazepine receptor inhibitors, cyclooxygenase-2 inhibitors and dihydrofolate reductase inhibitors.

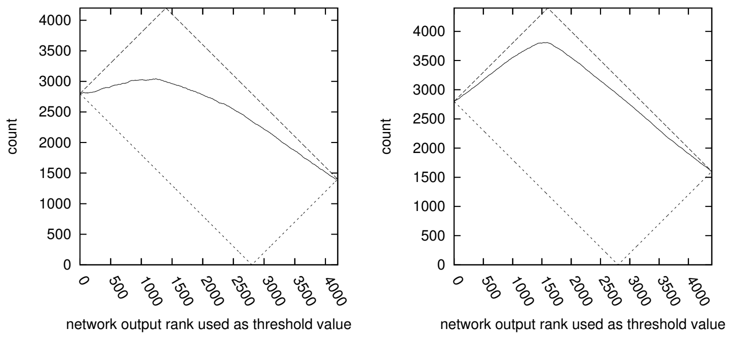

(a) BBB 20% CV (b) FXa 20% CV

Figure 2: A summary of performance on two classification cross validation experiments, InChI-NGN on blood brain barrier penetrating particles and factor Xa inhibitors.

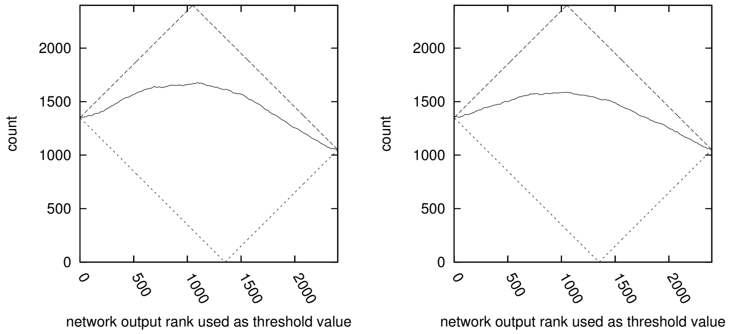

(a) Estrogen 20% CV, SMILES (b) Estrogen 20% CV, InChI

Figure 3: A summary of performance on two classification cross validation experiments, SMILES-NGN and InChI-NGN on estrogen receptor binders.

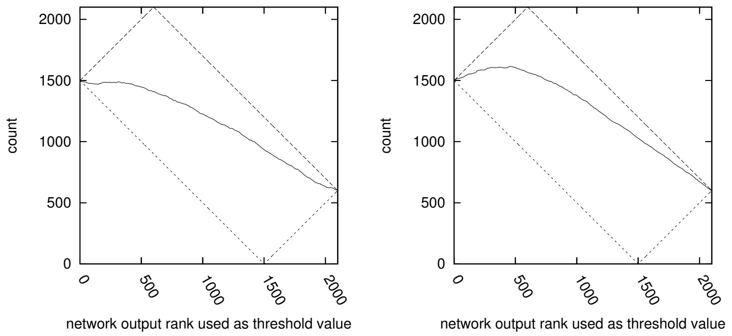

(a) Androgen 20% CV, SMILES (b) Androgen 20% CV, InChI

Figure 4: A summary of performance on two classification cross validation experiments, SMILES-NGN and InChI-NGN on androgen receptor binders.

SMILES-NGN toxicity prediction

We are providing to the community our complete dataset used for the SMILES-NGN toxicity prediction experiment. This document, supplementary2.pdf, contains a list of all molecules and LD50s with their respective sources.

Table 1 shows the results obtained when applying the ANN to the random training datasets. The first column represents the test subject. The second column contains the name of the organ implicated in the CPDB [1]. The third column represents one of 35 feature vectors that are implemented in the CDK [2]. Note that many feature vectors were unable to achieve convergence in the pre-testing phase and are therefore not included in the results. We show only the best feature vectors used with the ANN. The fourth column (Average/Epsilon) represents the average error between the ANN's predicted LD50 and the actual LD50 value over 100 trials and standard deviation of the error over 100 trials.

The fifth column measures the standard deviation of the Epsilon value on a per trial basis along with the standard deviation of that standard deviation. The final column shows the correlation coefficient for the know LD50 values to the estimates provided by ANN.

Table 2 contains the same information as Table 1 except for the per-trial, randomized training data.

Tables 3 and 4 show the data produced from this experiment with the use of the SMILES-NGN. Note, that the NGN does not use any descriptors, instead processing the entire molecule.

The final two tables represent how statistically relevant the data generated from this experiment are. Each method (ANN with all descriptor combinations and SMILES-NGN) were compared using a Wilcoxon signed-rank test. From the results, it is determined that each method belongs to a separate population where the average Epsilon and standard deviation are different. Comparison of those populations leads to the conclusion that one population is closer to zero than another. Table 5 shows the results for the grouped data, while Table 6 and Table 7 show the results from the random data. In each table, the animal, organ and descriptor used with the ANN are shown. The Wilcoxon's test statistic is shown for both the hypothesis that "Epsilon is lower for the NGN" and that "the standard deviation is lower for the NGN". A negative/positive value indicates the degree of support/lack-of-support for the hypothesis. A p-value of less than 0.05 indicates that the hypothesis is statistically significant at the 95% or 90% confidence interval.

From the group Wilcoxon test table it is shown that SMILES-NGN statistics may be lower than that of ANN, but it is not within a 95% or 90% confidence interval. By contrast for the random datasets, the

Table 1: Summary of the average values for trial epsilon (ϵ), trial standard deviation (σ), and correlation coefficient (ρ) of the random training by ANN with a variety of feature vectors

			Average±Standard Deviation		
Animal	Organ	Descriptor	ϵ	σ	ρ
Mouse	Kidney	Autocorrelation Charge	0.38 ± 0.12	0.23 ± 0.11	1 ± 0
		Autocorrelation Mass	0.31 ± 0.12	0.22 ± 0.14	0.98 ± 0.14
		Weighted Path	0.39 ± 0.15	0.26 ± 0.15	0.93 ± 0.25
	Lung	BCUT	0.18 ± 0.06	0.18 ± 0.06	0.15 ± 0.16
		Chi Path	0.2 ± 0.08	0.18 ± 0.08	0.16 ± 0.19
		Weighted Path	0.23 ± 0.08	0.22 ± 0.03	0.18 ± 0.17
	Stomach	BCUT	0.2 ± 0.08	0.13 ± 0.09	0.46 ± 0.33
		Chi Path	0.28 ± 0.09	0.18 ± 0.08	0.55 ± 0.37
		Weighted Path	0.26 ± 0.1	0.15 ± 0.09	0.54 ± 0.34
Rat	Kidney	Chi Path	0.17 ± 0.04	0.15 ± 0.04	0.18 ± 0.19
	Lung	ALOGP	0.27 ± 0.15	0.17 ± 0.14	0.74 ± 0.44
		Autocorrelation Charge	0.12 ± 0.12	0.12 ± 0.14	0.68 ± 0.47
		Autocorrelation Mass	0.31 ± 0.16	0.25 ± 0.12	0.42 ± 0.4
		Autocorrelation Polarizability	0.17 ± 0.09	0.11 ± 0.08	0.98 ± 0.14
		BCUT	0.12 ± 0.04	0.06 ± 0.04	0.99 ± 0.1
		Chi Path	0.16 ± 0.08	0.09 ± 0.07	1 ± 0
		Kappa Shape Indices	0.26 ± 0.17	0.23 ± 0.19	0.72 ± 0.45
		Weighted Path	0.2 ± 0.09	0.2 ± 0.13	0.38 ± 0.39
	Stomach	BCUT	0.2 ± 0.06	0.21 ± 0.05	0.17 ± 0.27
		Chi Cluster	0.14 ± 0.06	0.15 ± 0.09	0.35 ± 0.28
		Chi Path	0.15 ± 0.05	0.12 ± 0.05	0.39 ± 0.29
		Chi Path Cluster	0.2 ± 0.08	0.18 ± 0.06	0.37 ± 0.28
		Weighted Path	0.19 ± 0.06	0.16 ± 0.04	0.15 ± 0.19

Table 2: Summary of the average values for trial epsilon (ϵ), trial standard deviation (σ), and correlation coefficient (ρ) of the group training by ANN with a variety of feature vectors

			Average±Standard Deviation		
Animal	Organ	Descriptor	ϵ	σ	ρ
Mouse	Lung	BCUT	0.04 ± 0.04	0.18 ± 0.05	0.45 ± 0.07
		Chi Path	0.18 ± 0.09	0.17 ± 0.05	0.3 ± 0.3
		Weighted Path	0.15 ± 0.06	0.18 ± 0.13	0.02 ± 0.02
	Stomach	BCUT	0.15 ± 0.03	0.1 ± 0.05	0.59 ± 0.32
		Chi Path	0.24 ± 0.11	0.14 ± 0.11	0.39 ± 0.37
		Weighted Path	0.25 ± 0.08	0.25 ± 0.07	0.54 ± 0.34
Rat	Kidney	Chi Path	0.21 ± 0.06	0.23 ± 0.09	0.19 ± 0.19
	Lung	ALOGP	0.37 ± 0.22	0.24 ± 0.13	0.6 ± 0.55
		Autocorrelation Charge	0.17 ± 0.13	0.2 ± 0.19	0.54 ± 0.51
		Autocorrelation Mass	0.29 ± 0.15	0.33 ± 0.15	0.66 ± 0.41
		Kappa Shape Indices	0.31 ± 0.17	0.21 ± 0.18	0.7 ± 0.45
		Weighted Path	0.15 ± 0.07	0.17 ± 0.15	0.57 ± 0.47
	Stomach	BCUT	0.14 ± 0.04	0.12 ± 0.09	0.63 ± 0.34
		Chi Cluster	0.11 ± 0.06	0.14 ± 0.12	0.57 ± 0.41
		Chi Path	0.19 ± 0.09	0.15 ± 0.09	0.55 ± 0.24
		Weighted Path	0.2 ± 0.05	0.14 ± 0.03	0.27 ± 0.23

Table 3: Summary of the average values for trial epsilon (ϵ), trial standard deviation (σ), and correlation coefficient (ρ) of the random training by SMILES submitted to the NGN

		Average±Standard Deviation		
Animal	Organ	ϵ	σ	ρ
Mouse	Kidney	0.21 ± 0.11	0.14 ± 0.09	1 ± 0
	Lung	0.16 ± 0.05	0.15 ± 0.05	0.12 ± 0.14
	Stomach	0.2 ± 0.09	0.15 ± 0.09	0.48 ± 0.35
Rat	Kidney	0.18 ± 0.05	0.14 ± 0.04	0.11 ± 0.12
	Liver	0.17 ± 0.06	0.15 ± 0.06	0.14 ± 0.14
	Lung	0.12 ± 0.08	0.11 ± 0.09	0.48 ± 0.36
	Stomach	0.18 ± 0.06	0.12 ± 0.05	0.22 ± 0.24

Table 4: Summary of the average values for trial epsilon (ϵ), trial standard deviation (σ), and correlation coefficient (ρ) of the group training by SMILES submitted to the NGN

		Average±Standard Deviation		
Animal	Organ	ϵ	σ	ρ
Mouse	Lung	0.16 ± 0.06	0.17 ± 0.03	0.24 ± 0.13
	Stomach	0.16 ± 0.1	0.13 ± 0.09	0.56 ± 0.33
Rat	Kidney	0.19 ± 0.03	0.14 ± 0.04	0.07 ± 0.06
	Liver	0.15 ± 0.07	0.12 ± 0.07	0.26 ± 0.17
	Lung	0.09 ± 0.06	0.1 ± 0.08	0.82 ± 0.26
	Stomach	0.2 ± 0.07	0.14 ± 0.04	0.33 ± 0.43

NGN was consistently lower than the ANN for most descriptors within a 95% confidence interval, since p-values tend to be lower than 0.05.

Table 8 shows the best-performing method for each problem (broken down in terms of animal, organ, statistic, and training method). Results that were found to be statistically significant at the 95% confidence level are asterisked. This table shows that the SMILES-NGN outperforms all other methods.

References

1. Gold LS, Ames BN, Bernstein L, Blumenthal M, Chow K, Costa MD, de Veciana M, Eisenberg S, Garfinkel GB, Haggin T, Havender WR, Hooper NK, Levinson R, Lopipero P, Magaw R, Manley NB, MacLeod PM, Peto R, Pike MC, Rohrbach L, Sawyer CB, Slone TH, Smith M, Stern BR, Wong M: **Summary of Carcinogenic Potency Database by Target Organ** 2005, [http://potency.berkeley.edu/pathology.table.html]. [Date of Access May 2009].

2. Steinbeck C, Han Y, Kuhn S, Horlacher O, Luttmann E, Willighagen E: **The Chemistry Development Kit (CDK): An Open-Source Java Library for Chemo- and Bioinformatics**. *Journal of Chemical Information and Computer Sciences* 2003, **43**(2):493–500.

Table 5: Statistical results of Wilcoxon signed-rank test (null hypothesis of SMILES-NGN statistic is less than ANN with feature vectors, an alternative hypothesis that the true location shift is less than 0, and assumptions of unequal variations and no pairing between samples) for group training data(*95% Confidence Interval Reached)

Animal Organ Data Set Combination	Traditional Descriptor Being Compared to SMILES	Statistic	Test Statistic	p-value	90% Confidence Interval	Location Parameter
Mouse Lung	BCUT	Epsilon	5	0.5714	$(-Inf, 0.1205268)$	0.01499170
		Std Dev	4	0.4286	$(-Inf, 0.05418094)$	−0.02241334
	Chi Path	Epsilon	13	0.5794	$(-Inf, 0.0577229)$	0.003372274
		Std Dev	13	0.5794	$(-Inf, 0.04036957)$	0.005650336
	Weighted Path	Epsilon	5	0.5714	$(-Inf, 0.1386837)$	0.01702964
		Std Dev	5	0.5714	$(-Inf, 0.1120267)$	−0.02164081
Mouse Stomach	BCUT	Epsilon	11	0.4206	$(-Inf, 0.1225372)$	−0.01460621
		Std Dev	13	0.5794	$(-Inf, 0.1289036)$	0.03184872
	Chi Path	Epsilon	7	0.1548	$(-Inf, 0.05902415)$	−0.07127287
		Std Dev	14	0.6548	$(-Inf.0.09668496)$	0.009321175
	Weighted Path	Epsilon	5	0.0754	$(-Inf, 0.03941529)$	−0.08279434
		Std Dev	4	0.04762	$(-Inf, -0.03110177)$	−0.09498184
Rat Lung	ALOGP	Epsilon	1	0.007937	$(-Inf, -0.07625589)$	−0.2288930
		Std Dev	6	0.1111	$(-Inf, 0.03234261)$	−0.1532512
	Autocorrelation Charge	Epsilon	8	0.2103	$(-Inf, 0.03655133)$	−0.05918382
		Std Dev	7	0.1548	$(-Inf, 0.0989314)$	−0.02808754
	Autocorrelation Mass	Epsilon	3	0.02778	$(-Inf, -0.04183296)$	−0.1904236
		Std Dev	3	0.02778	$(-Inf, -0.07286496)$	−0.2644248
	Kappa Shape Indices	Epsilon	3	0.02778	$(-Inf, -0.02863745)$	−0.2022525
		Std Dev	8	0.2103	$(-Inf, 0.1036486)$	−0.128355
	Weighted Path	Epsilon	6	0.1111	$(-Inf, 0.02267382)$	−0.05934372
		Std Dev	10	0.3452	$(-Inf, 0.1146609)$	−0.05260102
Rat Kidney	Chi Path	Epsilon	5	0.1429	$(-Inf, 0.06070083)*$	−0.04736575
		Std Dev	4	0.09524	$(-Inf, 0.05366482)*$	−0.1082352
Rat Stomach	BCUT	Epsilon	20	0.9524	$(-Inf, 0.1137183)$	0.07935205
		Std Dev	18	0.8889	$(-Inf, 0.09154288)$	0.05501437
	Chi Cluster	Epsilon	17	0.9683	$(-Inf, 0.1601299)$	0.1352369
		Std Dev	13	0.7937	$(-Inf, 0.09786212)$	0.03980898
	Chi Path	Epsilon	12	0.5	$(-Inf, 0.1178148)$	−0.02121705
		Std Dev	14	0.6548	$(-Inf, 0.06871197)$	0.006018149
	Weighted Path	Epsilon	5	0.5714	$(-Inf, 0.0877907)$	0.005236316
		Std Dev	4	0.4286	$(-Inf, 0.05862452)$	−0.003071012

Table 6: Statistical results of Wilcoxon signed-rank test (null hypothesis of SMILES-NGN statistic is less than ANN with feature vectors, an alternative hypothesis that the true location shift is less than 0, and assumptions of unequal variations and no pairing between samples) for random training data(mouse only)

Animal Organ Data Set Combination	Traditional Descriptor Being Compared to SMILES	Statistic	Test Statistic	p-value	95% Confidence Interval	Location Parameter
Mouse Kidney	Autocorrelation Charge	Epsilon	465	$8.863e-12$	$(-Inf, -0.1286765)$	−0.1591443
		Std Dev	933	$1.492e-05$	$(-Inf, -0.05682528)$	−0.0846207
	Autocorrelation Mass	Epsilon	1415	$7.669e-06$	$(-Inf, -0.06925954)$	−0.1077996
		Std Dev	1600	0.0001677	$(-Inf, -0.04057833)$	−0.07627047
	Weighted Path	Epsilon	720	$2.010e-11$	$(-Inf, -0.1371728)$	−0.1692975
		Std Dev	1107	$4.676e-07$	$(-Inf, -0.08807012)$	−0.1344037
Mouse Lung	BCUT	Epsilon	519	0.1763	$(-Inf, 0.008936544)$	−0.01157805
		Std Dev	435	0.02875	$(-Inf, -0.002192690)$	−0.01972013
	Chi Path	Epsilon	1688	0.0006077	$(-Inf, -0.02028057)$	−0.03864846
		Std Dev	1864	0.005645	$(-Inf, -0.01017448)$	−0.03095166
	Weighted Path	Epsilon	157	0.0001426	$(-Inf, -0.03756032)$	−0.06536274
		Std Dev	81	$9.415e-07$	$(-Inf, -0.04588313)$	−0.06167551
Mouse Stomach	BCUT	Epsilon	2302	0.4621	$(-Inf, 0.02467575)$	−0.001496734
		Std Dev	2724	0.9546	$(-Inf, 0.05618853)$	0.02724963
	Chi Path	Epsilon	1253	$7.333e-06$	$(-Inf, -0.0492778)$	−0.07889718
		Std Dev	1892	0.06001	$(-Inf, 0.001435630)$	−0.02602904
	Weighted Path	Epsilon	662	0.001193	$(-Inf, -0.02736779)$	−0.06067118
		Std Dev	1051	0.5047	$(-Inf, 0.03347364)$	0.0001164592

Table 7: Statistical results of Wilcoxon signed-rank test (null hypothesis of SMILES-NGN statistic is less than ANN with feature vectors, an alternative hypothesis that the true location shift is less than 0, and assumptions of unequal variations and no pairing between samples) for random training data(rat only)

Animal Organ Data Set Combination	Traditional Descriptor Being Compared to SMILES	Statistic	Test Statistic	p-value	95% Confidence Interval	Location Parameter
Rat Kidney	Chi Path	Epsilon	1705	0.9195	$(-Inf, 0.02356395)$	0.01084519
		Std Dev	1355	0.2337	$(-Inf, 0.00857399)$	−0.006503034
Rat Lung	ALOGP	Epsilon	576	$3.13e-10$	$(-Inf, -0.09818032)$	−0.1313443)
		Std Dev	1293	0.01008	$(-Inf, -0.01230202)$	−0.04697898
	Autocorrelation Charge	Epsilon	2913	0.9504	$(-Inf, 0.03885386)$	0.02270924
		Std Dev	2675	0.758	$(-Inf, 0.02474942)$	0.007717613
	Autocorrelation Mass	Epsilon	749	$1.488e-12$	$(-Inf, -0.1239984)$	−0.1577959
		Std Dev	876	$4.82e-11$	$(-Inf, -0.1280000)$	−0.168888
	Autocorrelation Polarizability	Epsilon	1787	0.002854	$(-Inf, -0.01797089)$	−0.04064453
		Std Dev	2385	0.3595	$(-Inf, 0.01415508)$	−0.004771724
	BCUT	Epsilon	2270	0.1801	$(-Inf, 0.008510483)$	−0.009633803
		Std Dev	3110	0.9925	$(-Inf, 0.04492193)$	0.02350813
	Chi Path	Epsilon	1692	0.0006423	$(-Inf, -0.02117260)$	−0.04049815
		Std Dev	2819	0.8986	$(-Inf, 0.03032585)$	0.01228360
	Kappa Shape Indices	Epsilon	1283	$1.525e-06$	$(-Inf, -0.07393586)$	−0.1256972
		Std Dev	1704	0.001610	$(-Inf, -0.02269205)$	−0.09731576
	Weighted Path	Epsilon	1251	$3.221e-07$	$(-Inf, -0.05341745)$	−0.0769813
		Std Dev	1359	$2.722e-06$	$(-Inf, -0.04749109)$	−0.0956124
Rat Stomach	BCUT	Epsilon	464	0.09473	$(-Inf, 0.006444876)$	−0.02265008
		Std Dev	131	$6.954e-08$	$(-Inf, -0.07098777)$	−0.09547542
	Chi Cluster	Epsilon	1841	0.9974	$(-Inf, 0.05444701)$	0.03566873
		Std Dev	1234	0.1475	$(-Inf, 0.00842179)$	−0.01498036
	Chi Path	Epsilon	3247	0.9986	$(-Inf, 0.04711679)$	0.02936394
		Std Dev	2774	0.8631	$(-Inf, 0.02303095)$	0.008679174
	Chi Path Cluster	Epsilon	826	0.07944	$(-Inf, 0.003611578)$	−0.01963982)
		Std Dev	474	$9.904e-06$	$(-Inf, -0.03461324)$	−0.05646186
	Weighted Path	Epsilon	415	0.2121	$(-Inf, 0.01533738)$	−0.01372751
		Std Dev	288	0.006117	$(-Inf, -0.01463408)$	−0.03291495

Table 8: Best performing method for each problem type.

Animal	Organ	Statistic (Epsilon or Standard Deviation)	Training (Grouped or Random)	Best Method
Mouse	Kidney	Epsilon	Grouped	none
			Random	SMILES-NGN*
		Standard Deviation	Grouped	none
			Random	SMILES-NGN*
	Lung	Epsilon	Grouped	BCUT-ANN
			Random	SMILES-NGN
		Standard Deviation	Grouped	ChiPath-ANN or SMILES-NGN
			Random	SMILES-NGN*
	Stomach	Epsilon	Grouped	BCUT-ANN
			Random	SMILES-NGN
		Standard Deviation	Grouped	BCUT-ANN
			Random	BCUT-ANN*
Rat	Kidney	Epsilon	Grouped	SMILES-NGN
			Random	ChiPath-ANN
		Standard Deviation	Grouped	SMILES-NGN
			Random	SMILES-NGN
	Liver	Epsilon	Grouped	SMILES-NGN
			Random	SMILES-NGN
		Standard Deviation	Grouped	SMILES-NGN
			Random	SMILES-NGN
	Lung	Epsilon	Grouped	SMILES-NGN
			Random	BCUT-ANN or SMILES-NGN
		Standard Deviation	Grouped	SMILES-NGN
			Random	BCUT-ANN
	Stomach	Epsilon	Grouped	ChiCluster-ANN
			Random	ChiCluster-ANN*
		Standard Deviation	Grouped	BCUT-ANN
			Random	ChiPath-ANN or SMILES-NGN
